# Review on natural products databases: where to find data in 2020

**DOI:** 10.1186/s13321-020-00424-9

**Published:** 2020-04-03

**Authors:** Maria Sorokina, Christoph Steinbeck

**Affiliations:** University Friedrich-Schiller, Lessing Strasse 8, 07743 Jena, Germany

**Keywords:** Natural products, Databases, Traditional medicines, Drug discovery

## Abstract

Natural products (NPs) have been the centre of attention of the scientific community in the last decencies and the interest around them continues to grow incessantly. As a consequence, in the last 20 years, there was a rapid multiplication of various databases and collections as generalistic or thematic resources for NP information. In this review, we establish a complete overview of these resources, and the numbers are overwhelming: over 120 different NP databases and collections were published and re-used since 2000. 98 of them are still somehow accessible and only 50 are open access. The latter include not only databases but also big collections of NPs published as supplementary material in scientific publications and collections that were backed up in the ZINC database for commercially-available compounds. Some databases, even published relatively recently are already not accessible anymore, which leads to a dramatic loss of data on NPs. The data sources are presented in this manuscript, together with the comparison of the content of open ones. With this review, we also compiled the open-access natural compounds in one single dataset a COlleCtion of Open NatUral producTs (COCONUT), which is available on Zenodo and contains structures and sparse annotations for over 400,000 non-redundant NPs, which makes it the biggest open collection of NPs available to this date.

## Introduction

Natural products (NPs), are broadly defined as chemicals produced by living organisms. More precise definitions of NPs exist, but they do not always meet a consensus: some of the NPs include all small molecules that result from metabolic reactions, others classify as “NP” only products of secondary, or non-essential, metabolism. In this review, we made the choice to exclude molecules that participate in the primary, or essential, metabolism, such as energy or anabolic pathways, and consider only molecules that are produced by living organisms in order to accomplish a “higher” function, such as signalling or defence and still smaller than 1500 Da. However, as for most of the definitions in life sciences, the line between primary and secondary metabolites is very thin and depends on the potential application of the molecule to categorise This categorisation justifies the necessity of dedicated NP databases or a proper annotation in generalistic databases of molecules.

NPs have evolved over millions of years and acquired a unique chemical diversity, which consequently results in the diversity of their biological activities and drug-like properties. Therefore, even before the rise of the modern chemical pharmacology, NPs have been used for centuries as components of traditional medicines, in particular as active components of herbal remedies. Nowadays, some of the traditional healing practices, such as Indian Ayurveda, traditional Chinese medicine or African herbal medicines, remain the primary treatment option for many people across the world, due to economic reasons, to personal beliefs or to the difficulty in accessing pharmaceutical products. In modern pharmacology too, NPs have become one of the most important resources for developing new lead compounds and scaffolds [1–3]. Every week, scientific articles in peer-reviewed journals are published describing the positive effects of NPs on the healing process of various human and animal diseases. Major classes of antibiotics and antifungals are based on NPs isolated from microorganisms. Drugs used in the treatment of various cancers, cardiovascular diseases, diabetes, and more are often NPs or their derivatives. For instance, between 1981 and 2014 over 50% of newly developed drugs were developed from NPs [1]. NPs and their derivatives are also actively studied in food [4–8], cosmetic industries [9, 10] and in agriculture, with natural pesticides development [11]. This growing interest over NPs and their application resolved in uncontrollable growth of the number of published open and commercial databases, industrial catalogues, books of NPs and collections of structures provided in supplementary materials or research articles, compiling NPs from various organisms, geographical locations, targeted diseases and traditional uses. It became, therefore, a real challenge to find a complete and comprehensive open database for NPs. One other major problem is the publication of structures only in graphical format, such as in the annual reviews of Marine Natural Products [2]: these are not easily retrievable to be computationally analysed and they are not automatically integrated into public molecular databases. Virtual NP collections are therefore required for virtual screening, which is the first step in all exploratory molecular analyses and to some extent, in the discovery of NP-based drug or other types of active components. For example, the prior virtual screening of known NPs can prevent loss of time with extracting and purifying samples, postponing the wet lab step to the moment of theoretical identification of best candidates. In this way, the usage of modern cheminformatics technologies allows to accelerate research and save time and money for better results. The previous reviews on NPs databases are either outdated and do not reference the actual state of NP resources [12, 13], either focus on one particular type of application for such databases [14, 15], in particular databases that can be used for dereplication [16], a particular geographic origin of NPs [17] or simply do not refer a significant part of NP resources [18].

For this article, we reviewed a total of 123 resources listing NP structures cited in the scientific literature after 2000. Among them 92 are open and only 50 contain molecular structures that we could retrieve for analyses of their content, the overlap between them and compilation. The quality of molecular structures stored in these databases is also challenging: stereochemistry, for example, plays a major role in the function of NPs, and is the centre of a lot of research projects in the field. Despite this known importance, almost 12% of the collected molecules lack information on stereochemistry while having stereocenters. Finally, the non-redundant collection of NPs from these open resources has been assembled in a MongoDB COlleCtion of Open Natural prodUcTs (COCONUT).

## Natural products online resources: availability and characteristics

For now, there is no globally accepted community resource for NPs, where their structures and annotations can be submitted, edited and queried by a large public, like there is UniProt [19] for proteins or NCBI Taxonomy [20] for the classification of living organisms. This leads to an impressive (123) amount of various, open and commercial, with different scope and differently structured resources for NP structures and their annotations. Mentions of NP databases, datasets and collections across publications from 2000 to 2019 and in omicX [21], a catalogue of scientific databases and software, were collected and are listed in Table 1 [22].

**Table 1:** List of Natural Products databases cited in scientific literature since 2000. The list is ordered by alphabetical order of the database names, and contains, when available, extended metadata

Database name	NP type	Estimated size (number of NP molecules with correct structures)	Number of unique molecules in COCONUT	Percentage of molecules with stereochemistry	Is open (data can be freely browsed)	Recquires a registration	Is maintained (2019)	Is updated
3DMET	Generalistic	18248	x	x	Yes	No	Yes	Yes
AfroCancer	tm, plants, africa	390	365	69.76%	Yes	NA	NA	No
AfroDB	tm, plants, africa	954	874	70.73%	Yes	No	No	No
AfroMalariaDB	tm, plants, africa	265	252	70.93%	yes	NA	NA	no
Afrotryp	tm, plants, drug-like, africa	321	x	x	Unknown	NA	NA	No
Alkamid database	plants, structure	300	x	x	yes	no	yes	no
Ambinter-Greenpharma natural compound library (GPNCL)	Generalistic, industrial	> 150,000	x	x	No	Yes	Yes	Yes
AnalytiCon Discovery MEGx	bacteria, plants, industrial	5147	4908	44.15%	Yes	Yes	Yes	Yes
AntiBase	drug-like	> 40,000	x	x	No	No	Yes	No
AntiMarin	Marine, drug-like	> 60,000	x	x	No	Unknown	No	No
ATBD (Animal Toxin Database)	toxins	1000	x	x	Unknown	Unknown	No	Unknown
Ayurveda	tm, asia	950	x	x	No	Yes	Yes	Unknown
Berdy’s Bioactive Natural Products Database	Generalistic	x	x	x	No	Unknown	No	No
BiGG	Metabolites	7339	x	x	Yes	No	Yes	Yes
Binding DB	Drug-like	x	x	x	Yes	No	Yes	No
BIOFAQUIM	Plant, fungi, america	420	400	59.05%	Yes	No	Yes	Yes
BioPhytMol	Drug-like, plants, asia	633	x	x	Yes	No	Yes	Yes
BitterDB	Food	654	631	14.17%	Yes	No	Yes	Yes
BRENDA	Metabolites	x	x	x	Yes	No	Yes	Yes
CamMedNP	tm, plants, africa	> 2500	x	x	Yes, but proprietary format	No	NA	No
Carotenoids Database	Structure	1174	991	57.63%	Yes	No	Yes	Yes
CAS registry/SciFinder	Chemicals	> 300,000	x	x	No	Yes	Yes	Yes
CEMTDD - Chinese Ethnic Minority Traditional Drug Database	tm, plants, asia	4060	x	x	Yes	No	Yes	No
CHDD (Chinese Traditional Medicinal Herbs database)	tm, plants, asia	> 30,000	x	x	Unknown	Unknown	No	No
ChEBI	Chemicals	15,736	14,621	71.33%	Yes	No	Yes	Yes
Chem-TCM	Plants, tm, asia	> 12,000	x	x	No	Yes	Yes	No
ChemBank	Chemicals	x	x	x	Yes	No	No	No
ChEMBL	Chemicals	1899	1581	91.59%	Yes	No	Yes	Yes
ChemBridge diversity datasets	Generalistic, industrial	x	x	x	No	Yes	No	No
ChemDB	Plants, asia	> 1000	x	x	Unknown	Unknown	No	No
ChemIDplus	Drug-like, toxins	9042	x	x	Yes	No	Yes	Yes
ChemSpider	Chemicals	9732	9029	29.50%	Yes	No	Yes	Yes
CHMIS-C	Plants, tm, asia	> 8000	x	x	Yes	No	No	No
CMAUP	Plants	47,645	20,873	72.37%	Yes	No	Yes	No
CNPD (Chinese Natural Products Database)	Generalistic	> 57,000	x	x	Unknown	Unknown	No	No
ConMedNP	Plants, tm, africa	3118	2504	69.59%	Yes	NA	NA	NA
CSLS/NCI (Chemical Structure Lookup Service)	Metabolites	x	x	x	Yes	No	Yes	No
Database of Indonesian Medicinal Plants	Plants, tm, asia	6776	x	x	Yes	No	Yes	No
DESMSCI (Dragon Exploration System on Marin Sponge Compounds Interactions)	Marine	x	x	x	Yes	No	No	No
DFC (Dictionary of Food COmpounds)	Food	> 41,000	x	x	No	Yes	Yes	Yes
DMNP (Dictionary of Marine Natural Products)	Marine	> 30,000	x	x	No	Yes	Yes	Yes
DNP (Dictionary of Natural Products) by Chapman and Hall (also known as CHEMnetBase)	Generalistic	> 230,000	x	x	No	Yes	Yes	Yes
Drugbank NPs	Drug-like	2617	2617	51.32%	Yes	No	Yes	Yes
eBasis	Food		x	x	No	Yes	Yes	Yes
ETCM (Encyclopedia of Traditional Chinese Medicine)	tm, asia	7274	x	x	Yes	No	Yes	Yes
ETM-DB	tm, plants, africa	1795	1653	40.46%	Yes	No	Yes	Yes
FooDB	Food	24,215	22,223	36.01%	Yes	No	Yes	Yes
GNPS	Dereplication	7619	6708	31.08%	Yes	No	Yes	Yes
HIM (Herbal Ingridients in-vivo Metabolism database)	Drug-like, tm, plants	1261	962	41.62%	Yes	No	No	No
HIT (Herbal Ingridients Targets)	Drug-like, tm, plants	524	472	44.03%	Yes	No	No	No
HMDB	Dereplication	x	x	x	Yes	No	Yes	Yes
IMPPAT	tm, plants, asia	9596	x	x	Yes	No	Yes	Yes
InflamNat	Drug-like	552	536	63.75%	Yes	NA	NA	NA
Indofine Chemical Company Inc. natural products	Generalistic, industrial	56	46	51.06%	Yes	No	Yes	
InPACdb	Drug-like, plants, asia	124	121	62.10%	Yes	No	No	No
InterBioScreen Ltd (IBS)	Generalistic, industrial	68,350	67,292	42.17%	Yes	Yes	Yes	Yes
iSMART	tm, plants, asia	x	x	x	Yes	No	Yes	Yes
KEGG	Metabolites	x	x	x	Yes	No	Yes	Yes
KNApSaCK	Plants	10,265	8887	74.76%	Yes	No	Yes	Yes
Lichen Database	Fungi	249	156	26.67%	Yes	No	Yes	No
LOPAC1280 by Merck	Drug-like	1280	x	x	No	Yes	Yes	Yes
MAPS database	Plants, asia	x	x	x	Unknown	Unknown	No	No
Marine Compound Database (MCDB)	Marine	182	x	x	Yes	No	No	No
Marine Natural Product Database (MNPD)	Marine	6000	x	x	Yes	No	No	No
MarineLit	Marine	> 29,000	x	x	No	Yes	Yes	Yes
Massbank	Dereplication	x	x	x	Yes	No	Yes	Yes
MedPServer	Plants, tm, asia, drug-like	1124	x	x	Yes	No	Yes	Yes
MetaCyc	Metabolites	x	x	x	Yes	No	Yes	Yes
METLIN	Dereplication	x	x	x	Yes	Yes	Yes	Yes
Mitishamba database	Plants, africa	1102	1010	23.84%	Yes	No	Yes	No
NADI	tm, plants	3000	x	x	No	Yes	Yes	Unknown
NANPDB	Plants, africa	6832	3913	75.02%	Yes	No	Yes	Yes
NaprAlert	Generalistic	> 15,5000	x	x	No	Yes	Yes	Yes
NAPROC-13	Dereplication	> 18,000	x	x	Yes	No	Yes	Yes
NCI DTP data	Drug-like	418	404	36.76%	Yes	No	Yes	No
NeMedPlant	tm, plants, asia	100	x	x	Yes	No	Yes	No
NIST	Chemicals	x	x	x	No	No	Yes	Yes
NMRDATA	Dereplication	x	x	x	Unknown	Yes	Yes	Yes
NMRShiftDB	Dereplication	1875	x	x	Yes	No	Yes	Yes
Novel Antibiotics database	Drug-like	5430	x	x	Yes	No	Yes	No
NPACT	Plants, drug-like	1573	1453	77.53%	Yes	No	Yes	Yes
NPASS	Plants, bacteria, metazoa, fungi	30,858	27,479	71.58%	Yes	No	Yes	Yes
NPAtlas	Bacteria, fungi	20,035	18,959	67.03%	Yes	No	Yes	Yes
NPCARE	Plants, marine, bacteria, drug-like	1370	1364	0%	Yes	No	Yes (server fails sometimes)	Yes
NPEdia	Generalistic	18,016	16,190	51.83%	Yes	No	Yes	No
NPL (library)	Plants, drug-like	814	x	x	No	NA	NA	NA
NuBBEDB	Plants, insects, america	2215	2022	58.34%	Yes	No	Yes	
Open Source Malaria	Drug-like	842	x	x	Yes	No	Yes	Yes
p-ANAPL (Pan-African Natural Product Library )	Plants, africa	538	467	0.86%	Yes	No	NA	
PAMDB	Metabolites, bacteria	x	x	x	Yes	No	Yes	Yes
Phenol-explorer	Food	862	681	51.79%	Yes	No	Yes	NA
Phytochemica	Plants, tm, asia	571	x	x	Yes	No	No	No
PhytoHub	Food, plants	1200	x	x	Yes	No	Yes	Yes
Pi Chemicals System Natural Products	Generalistic, industrial	405	x	x	Yes	No	Yes	Yes
Prestwick	Plants, industrial	320	x	x	No	Yes	Yes	Yes
ProCarDB	Structure, bacteria	304	x	x	Yes	No	Yes	No
PubChem	Chemicals	3529	2835	2.33%	Yes	No	Yes	Yes
REAXYS	Chemicals	> 220,000	x	x	No	Yes	Yes	
ReSpect	Dereplication	4767	711	0%	Yes	No	Yes	No
SANCDB	Plants, africa	623	592	82.28%	Yes	No	Yes	Yes
Seaweed Metabolite Database (SWMD)	Marine	1110	423	78.53%	Yes	No	Yes	No
Specs Natural Products	Generalistic, industrial	745	745	53.02%	Yes	Yes	Yes	No
Spektraris NMR	Dereplication	248	242	91.53%	Yes	No	Yes	No
StreptomeDB	Bacteria	6415	3610	56.41%	Yes	No	Yes	No
Super Natural II	Generalistic	320,670	235,436	83.55%	Yes	No	Yes	No
Super Scent	Other	2100	x	x	Yes	No	Yes	No
Super Sweet	Food, metabolites	15,000	x	x	Yes	No	Yes	No
TargetMol Natural Compound Library	Generalistic, industrial	1680	x	x	No	Yes	Yes	Yes
TC-MC	tm, asia, plants	> 20,000	x	x	Yes	No	Yes	Yes
TCMDB@Taiwan	tm, asia, plants	58,351	50,891	90.38%	Yes	No	Yes (server fails sometimes)	Yes
TCMID	tm, asia, plants	12,549	10,572	0%	Yes	No	Yes	No
TCMSP	tm, asia, plants	29,384	x	x	Yes	No	No	No
TIM	tm, asia, plants	1829	x	x	No	Unknown	No	No
TIPdb	Asia, plants, drug-like	8656	7752	77.10%	Yes	No	Yes	No
TMDB	Plants, metabolites	1393	x	x	Yes	No	No	No
TPPT	Plants, toxins, europe	1583	1486	76.84%	Yes	No	Yes	No
TriForC	Plants	266	x	x	Yes	No	Yes	No
UEFS	Plants, america	503	481	68.93%	Yes	No	No	No
UNPD (Universal Natural Products Database)	Generalistic	213,100	156,984	12.62%	Yes	No	No	No
VIETHERB	Plants, asia	10,887	x	x	Yes	Unknown	No	No
Yeast Metabolome Database	Metabolites, dereplication	16,042	x	x	Yes	No	Yes	Yes
YaTCM	tm, asia, plants	47,696	x	x	Yes	No	Yes	Yes
ZINC natural products catalogue	Generalistic	85,198	673,36	90.49%	Yes	No	Yes	Yes

Database name	NP type	Estimated size (number of NP molecules with correct structures)	Is commercial	Molecule structures easily retrievable (download link, data packed in one file, bulk download option)	Has extensive metadata (organism, tissue, geo info, …)	Has cross-refs	Most recent publication (citation)	Most recent publication (DOI)
3DMET	Generalistic	18248	No	No	No	Yes	Maeda MH, Kondo K. Three-dimensional structure database of natural metabolites (3DMET): a novel database of curated 3D structures. J Chem Inf Model. 2013 Mar 25;53(3):527–33. 10.1021/ci300309k. Epub 2013 Mar 7. PubMed PMID: 23293959.	10.1021/ci300309k
AfroCancer	tm, plants, africa	390	No	NA	No	No	Fidele Ntie-Kang, Justina Ngozi Nwodo, Akachukwu Ibezim, Conrad Veranso Simoben, Berin Karaman, Valery Fuh Ngwa, Wolfgang Sippl, Michael Umale Adikwu, Luc Meva’a Mbaze, “Molecular Modeling of Potential Anticancer Agents from African Medicinal Plants”, J. Chem. Inf. Model.20145492433-2450	10.1021/ci5003697
AfroDB	tm, plants, africa	954	No	Yes	No	No	Ntie-Kang F, Zofou D, Babiaka SB, Meudom R, Scharfe M, Lifongo LL, et al. (2013) AfroDb: A Select Highly Potent and Diverse Natural Product Library from African Medicinal Plants. PLoS ONE 8(10): e78085.	10.1371/journal.pone.0078085
AfroMalariaDB	tm, plants, africa	265	no	NA	no	no	Onguéné, P.A., Ntie-Kang, F., Mbah, J.A. et al. “The potential of anti-malarial compounds derived from African medicinal plants, part III: an in silico evaluation of drug metabolism and pharmacokinetics profiling”, Org Med Chem Lett (2014) 4: 6.	10.1186/s13588-014-0006-x
Afrotryp	tm, plants, drug-like, africa	321	No	NA	Unknown	Unknown	Ibezim, A., Debnath, B., Ntie-Kang, F. et al. “Binding of anti-Trypanosoma natural products from African flora against selected drug targets: a docking study” Med Chem Res (2017) 26: 562. 10.1007/s00044-016-1764-y	10.1007/s00044-016-1764-y
Alkamid database	plants, structure	300	no	no	yes	no	Boonen, J., Bronselaer, A., Nielandt, J., Veryser, L. De Tré, G., De Spiegeleer, B., 2012. Alkamid database: Chemistry, occurrence and functionality of plant N-alkylamides. Journal of Ethnopharmacology 2012; 142(3): 563–590	10.1016/j.jep.2012.05.038
Ambinter-Greenpharma natural compound library (GPNCL)	Generalistic, industrial	> 150,000	Yes	No	Unknown	Unknown	x	x
AnalytiCon Discovery MEGx	bacteria, plants, industrial	5147	No	Yes	No	No	x	x
AntiBase	drug-like	> 40,000	Yes	Yes	Unknown	Unknown	Laatsch H (2012) Antibase Version 4.0—The natural compound identifier. Wiley-VCH Verlag GmbH & Co. KGaA	x
AntiMarin	Marine, drug-like	> 60,000	Yes	Unknown	Unknown	Unknown	Blunt, J., Munro, M. & Laatsch, H. Antimarin database. University of Canterbury; Christchurch, New Zealand: University of Gottingen; Gottingen, Germany, (2007).	x
ATBD (Animal Toxin Database)	toxins	1000	No	Unknown	Unknown	Unknown	He QY, He QZ, Deng XC, et al. ATDB: a uni-database platform for animal toxins. Nucleic Acids Res. 2008;36(Database issue):D293–D297. 10.1093/nar/gkm832	10.1093/nar/gkm832
Ayurveda	tm, asia	950	Unknown	Unknown	Unknown	Unknown	Lagunin AA, Druzhilovsky DS, Rudik AV, Filimonov DA, Gawande D, Suresh K, Goel R, Poroikov VV. [Computer evaluation of hidden potential of phytochemicals of medicinal plants of the traditional Indian ayurvedic medicine]. Biomed Khim. 2015 Mar-Apr;61(2):286–97. 10.18097/pbmc20156102286. Russian. PubMed PMID:25978395.	10.18097/PBMC20156102286
Berdy’s Bioactive Natural Products Database	Generalistic	x	Yes	Unknown	Unknown	Unknown	Berdy J., Kertesz M. (1989) Bioactive natural products database: an aid for natural products identification. In: Collier H.R. (eds) Chemical Information. Springer, Berlin, Heidelberg	10.1007/978-3-642-75165-3_23
BiGG	Metabolites	7339	No	NA	Yes	Yes	King ZA, Lu JS, Dräger A, Miller PC, Federowicz S, Lerman JA, Ebrahim A, Palsson BO, and Lewis NE. BiGG Models: A platform for integrating, standardizing, and sharing genome-scale models (2016) Nucleic Acids Research 44(D1):D515-D522.	10.1093/nar/gkv1049
Binding DB	Drug-like	x	No	Yes	Yes	Yes	Gilson MK, Liu T, Baitaluk M, Nicola G, Hwang L, Chong J. BindingDB in 2015: A public database for medicinal chemistry, computational chemistry and systems pharmacology. Nucleic Acids Res. 2016;44(D1):D1045–D1053.	10.1093/nar/gkv1072
BIOFAQUIM	Plant, fungi, america	420	No	Yes	Yes	No	Pilón-Jiménez, B.A.; Saldívar-González, F.I.; Díaz-Eufracio, B.I.; Medina-Franco, J.L. BIOFACQUIM: A Mexican Compound Database of Natural Products. Biomolecules 2019, 9, 31	10.3390/biom9010031
BioPhytMol	Drug-like, plants, asia	633	No	No	Yes	No	Sharma A, Dutta P, Sharma M, Rajput NK, Dodiya B, Georrge JJ, Kholia T; OSDD Consortium, Bhardwaj A. BioPhytMol: a drug discovery community resource on anti-mycobacterial phytomolecules and plant extracts. J Cheminform. 2014 Oct 11;6(1):46. eCollection 2014 Dec. PubMed PMID: 25360160; PubMed Central PMCID: PMC4206768.	10.1186/s13321-014-0046-2
BitterDB	Food	654	No	No	Yes	Yes	Ayana Dagan Wiener, Antonella Di Pizio, Ido Nissim, Malkeet Singh Bahia; Nitzan Dubovski, Eitan Margulis, Masha Y. Niv. BitterDB: Taste ligands and receptors database in 2019. Nucleic Acids Res 2019: gky974.	10.1093/nar/gky974
BRENDA	Metabolites	x	No	Yes	Yes	Yes	Jeske L, Placzek S, Schomburg I, Chang A, Schomburg D. BRENDA in 2019: a European ELIXIR core data resource. Nucleic Acids Res. 2019;47(D1):D542–D549. doi:10.1093/nar/gky1048	10.1093/nar/gky1048
CamMedNP	tm, plants, africa	> 2500	No	Yes, but proprietary format (MDB readable by MOE)	Unknown	Unknown	Ntie-Kang F, Mbah JA, Mbaze LM, et al. CamMedNP: building the Cameroonian 3D structural natural products database for virtual screening. BMC Complement Altern Med. 2013;13:88. Published 2013 Apr 16. doi:10.1186/1472-6882-13-88	10.1186/1472-6882-13-88
Carotenoids Database	Structure	1174	No	Yes	Yes	Yes	Junko Yabuzaki, Carotenoids Database: structures, chemical fingerprints and distribution among organisms, Database, Volume 2017, 2017, bax004	10.1093/database/bax004
CAS registry/SciFinder	Chemicals	> 300,000	Yes	Unknown	Unknown	Unknown	Gabrielson SW. SciFinder. J Med Libr Assoc. 2018;106(4):588–590. doi:10.5195/jmla.2018.515	10.5195/jmla.2018.515
CEMTDD - Chinese Ethnic Minority Traditional Drug Database	tm, plants, asia	4060	No	No	Yes	No	Huang J, Zheng Y, Wu W, et al. CEMTDD: The database for elucidating the relationships among herbs, compounds, targets and related diseases for Chinese ethnic minority traditional drugs. Oncotarget. 2015;6(19):17675–17684	10.18632/oncotarget.3789
CHDD (Chinese Traditional Medicinal Herbs database)	tm, plants, asia	> 30,000	Unknown	Unknown	Unknown	Unknown	Xuebin Qiao, Tingjun Hou, Wei Zhang, SenLi Guo, Xiaojie Xu, “A 3D Structure Database of Components from Chinese Traditional Medicinal Herbs”, J. Chem. Inf. Comput. Sci.2002423481-489	10.1021/ci010113h
ChEBI	Chemicals	15,736	No	Yes	Yes	Yes	Janna Hastings, Gareth Owen, Adriano Dekker, Marcus Ennis, Namrata Kale, Venkatesh Muthukrishnan, Steve Turner, Neil Swainston, Pedro Mendes, Christoph Steinbeck, ChEBI in 2016: Improved services and an expanding collection of metabolites, Nucleic Acids Research, Volume 44, Issue D1, 4 January 2016, Pages D1214–D1219	10.1093/nar/gkv1031
Chem-TCM	Plants, tm, asia	> 12,000	Yes	Unknown	Unknown	Unknown	Ehrman, T.; Barlow D.; Hylands, P. In silico search for multi-target anti-inflammatories in Chinese herbs and formulas. J Bioorganic & Med Chem. 2010, 18, Pages 2204–2218. doi:10.1016/j.bmc.2010.01.070	10.1016/j.bmc.2010.01.070
ChemBank	Chemicals	x	No	Unknown	Unknown	Unknown	Petri Seiler K, Kuehn H, Pat Happ M, Decaprio D, Clemons PA. Using ChemBank to probe chemical biology. Curr Protoc Bioinformatics. 2008 Jun;Chapter 14:Unit 14.5. 10.1002/0471250953.bi1405s22. Review. PubMed PMID: 18551413.	10.1002/0471250953.bi1405s22
ChEMBL	Chemicals	1899	No	Yes	No	Yes	Anna Gaulton, Anne Hersey, Michał Nowotka, A. Patrícia Bento, Jon Chambers, David Mendez, Prudence Mutowo, Francis Atkinson, Louisa J. Bellis, Elena Cibrián-Uhalte, Mark Davies, Nathan Dedman, Anneli Karlsson, María Paula Magariños, John P. Overington, George Papadatos, Ines Smit, Andrew R. Leach, The ChEMBL database in 2017, Nucleic Acids Research, Volume 45, Issue D1, January 2017, Pages D945–D954	10.1093/nar/gkw1074
ChemBridge diversity datasets	Generalistic, industrial	x	No	Unknown	Unknown	Unknown	x	x
ChemDB	Plants, asia	> 1000	No	Unknown	Unknown	Unknown	Mirza SB, Bokhari H, Fatmi MQ. Exploring Natural Products from the Biodiversity of Pakistan for Computational Drug Discovery Studies: Collection, Optimization, Design and Development of A Chemical Database (ChemDP). Curr Comput Aided Drug Des. 2015;11(2):102–9. PubMed PMID: 26343150.	10.2174/157340991102150904101740
ChemIDplus	Drug-like, toxins	9042	No	No	No	Yes	Patricia Tomasulo (2002) ChemIDplus-Super Source for Chemical and Drug Information, Medical Reference Services Quarterly, 21:1, 53–59	10.1300/J115v21n01_04
ChemSpider	Chemicals	9732	No	Yes	No	Yes	Harry E. Pence, Antony Williams, “ChemSpider: An Online Chemical Information Resource”, J. Chem. Educ.201087111123-1124	10.1021/ed100697w
CHMIS-C	Plants, tm, asia	> 8000	No	Unknown	Unknown	Unknown	Xueliang Fang, Lei Shao, Hui Zhang, Shaomeng Wang, “CHMIS-C: A Comprehensive Herbal Medicine Information System for Cancer”, J. Med. Chem.20054851481-1488	10.1021/jm049838d
CMAUP	Plants	47,645	No	Yes	Yes	Yes	Xian Zeng, Peng Zhang, Yali Wang, et al. CMAUP: a database of collective molecular activities of useful plants. Nucleic Acids Research 2019; 47(D1): D1118-D1127	10.1093/nar/gky965
CNPD (Chinese Natural Products Database)	Generalistic	> 57,000	Unknown	Unknown	Unknown	Unknown	Shen, Jianhua; Xu, Xiaoying; Cheng, Feng; Liu, Hong; Luo, Xiaomin; Shen, Jingkang; Chen, Kaixian; Zhao, Weimin; Shen, Xu; Jiang, Hualiang, “Virtual Screening on Natural Products for Discovering Active Compounds and Target Information”, Current Medicinal Chemistry, Volume 10, Number 21, 2003, pp. 2327–2342(16)	10.2174/0929867033456729
ConMedNP	Plants, tm, africa	3118	NA	NA	No	No	Fidele Ntie-Kang, Pascal Amoa Onguéné, Michael Scharfe, Luc C. Owono Owono, Eugene Megnassan, Luc Meva’a Mbaze, Wolfgang Sippl, Simon M. N. Efange, “ConMedNP: a natural product library from Central African medicinal plants for drug discovery”, RSC Adv., 2014, 4, 409–419	10.1039/C3RA43754J
CSLS/NCI (Chemical Structure Lookup Service)	Metabolites	x	No	Yes	No	Yes	M. Sitzmann, I.V. Filippov & M.C. Nicklaus (2008) Internet resources integrating many small-molecule databases1,SAR and QSAR in Environmental Research, 19:1–2, 1–9	10.1080/10629360701843540
Database of Indonesian Medicinal Plants	Plants, tm, asia	6776	No	No	No	No	Yanuar A, Mun’im A, Lagho ABA, Syahdi RR, Rahmat M, Suhartanto H. Medicinal plants database and three dimensional structure of the chemical compounds from medicinal plants in Indonesia. Int J Comput Sci. 2011;8:180–3.	https://arxiv.org/abs/1111.7183
DESMSCI (Dragon Exploration System on Marin Sponge Compounds Interactions)	Marine	x	No	No	Unknown	Unknown	Sagar S, Kaur M, Radovanovic A, Bajic VB. Dragon exploration system on marine sponge compounds interactions. J Cheminform. 2013 Feb 16;5(1):11. 10.1186/1758-2946-5-11. PubMed PMID: 23415072; PubMed Central PMCID: PMC3608955.”10.1186/1758-2946-5-11	10.1186/1758-2946-5-11
DFC (Dictionary of Food COmpounds)	Food	> 41,000	Yes	Yes	Unknown	Unknown	x	x
DMNP (Dictionary of Marine Natural Products)	Marine	> 30,000	Yes	Yes	Unknown	Unknown	x	x
DNP (Dictionary of Natural Products) by Chapman and Hall (also known as CHEMnetBase)	Generalistic	> 230,000	Yes	Yes	Unknown	Unknown	x	x
Drugbank NPs	Drug-like	2617	No	Yes	Yes	Yes	David S Wishart, Yannick D Feunang, An C Guo, Elvis J Lo, Ana Marcu, Jason R Grant, Tanvir Sajed, Daniel Johnson, Carin Li, Zinat Sayeeda, Nazanin Assempour, Ithayavani Iynkkaran, Yifeng Liu, Adam Maciejewski, Nicola Gale, Alex Wilson, Lucy Chin, Ryan Cummings, Diana Le, Allison Pon, Craig Knox, Michael Wilson, DrugBank 5.0: a major update to the DrugBank database for 2018, Nucleic Acids Research, Volume 46, Issue D1, 4 January 2018, Pages D1074–D1082	10.1093/nar/gkx1037
eBasis	Food		Yes	Unknown	Unknown	Unknown	J. Plumb, S. Pigat, F Bompola, M, Cushen, H Pinchen, E Nørby, S Astley, J Lyons, M Kiely and P Finglas. eBASIS (Bioactive Substances in Food Information Systems) and Bioactive Intakes: Major Updates of the Bioactive Compound Composition and Beneficial Bioeffects Database and the Development of a Probabilistic Model to Assess Intakes in Europe. Nutrients 2017, 9(4), 320	10.3390/nu9040320
ETCM (Encyclopedia of Traditional Chinese Medicine)	tm, asia	7274	No	No	Yes	No	Xu HY, Zhang YQ, Liu ZM, Chen T, Lv CY, Tang SH, Zhang XB, Zhang W, Li ZY, Zhou RR, Yang HJ, Wang XJ, Huang LQ. ETCM: an encyclopaedia of traditional Chinese medicine. Nucleic Acids Res. 2018 Oct 26.	10.1093/nar/gky987
ETM-DB	tm, plants, africa	1795	No	No	Yes	Yes	Bultum LE, Woyessa AM, Lee D. ETM-DB: integrated Ethiopian traditional herbal medicine and phytochemicals database. BMC Complement Altern Med. 2019 Aug 14;19(1):212. 10.1186/s12906-019-2634-1. PubMed PMID: 31412866; PubMed Central PMCID: PMC6692943.	10.1186/s12906-019-2634-1
FooDB	Food	24,215	No	Yes	Yes	Yes	x	x
GNPS	Dereplication	7619	No	Yes	No	No	Want et al. “Sharing and community curation of mass spectrometry data with Global Natural Products Social Molecular Networking”, Nature Biotechnology volume 34, pages 828–837 (2016)	10.1038/nbt.3597
HIM (Herbal Ingridients in-vivo Metabolism database)	Drug-like, tm, plants	1261	No	Unknown	Unknown	Unknown	Kang H, Tang K, Liu Q, Sun Y, Huang Q, Zhu R, Gao J, Zhang D, Huang C, Cao Z. HIM-herbal ingredients in-vivo metabolism database. J Cheminform. 2013 May 31;5(1):28. 10.1186/1758-2946-5-28. PubMed PMID: 23721660; PubMed Central PMCID: PMC3679852.	10.1186/1758-2946-5-28
HIT (Herbal Ingridients Targets)	Drug-like, tm, plants	524	No	Unknown	Unknown	Unknown	Hao Ye, Li Ye, Hong Kang, Duanfeng Zhang, Lin Tao, Kailin Tang, Xueping Liu, Ruixin Zhu, Qi Liu, Y. Z. Chen, Yixue Li, Zhiwei Cao, HIT: linking herbal active ingredients to targets, Nucleic Acids Research, Volume 39, Issue suppl_1, 1 January 2011, Pages D1055–D1059	10.1093/nar/gkq1165
HMDB	Dereplication	x	No	Yes	Yes	Yes	Wishart DS, Feunang YD, Marcu A, Guo AC, Liang K, Vázquez-Fresno R, Sajed T, Johnson D, Li C, Karu N, Sayeeda Z, Lo E, Assempour N, Berjanskii M, Singhal S, Arndt D, Liang Y, Badran H, Grant J, Serra-Cayuela A, Liu Y, Mandal R, Neveu V, Pon A, Knox C, Wilson M, Manach C, Scalbert A. HMDB 4.0: the human metabolome database for 2018. Nucleic Acids Res. 2018 Jan 4;46(D1):D608-D617. 10.1093/nar/gkx1089. PubMed PMID: 29140435; PubMed Central PMCID: PMC5753273.	10.1093/nar/gkx1089
IMPPAT	tm, plants, asia	9596	No	No	Yes	Yes	Mohanraj K, Karthikeyan BS, Vivek-Ananth RP, Chand RPB, Aparna SR, Mangalapandi P, Samal A. IMPPAT: A curated database of Indian Medicinal Plants, Phytochemistry And Therapeutics. Sci Rep. 2018 Mar 12;8(1):4329. 10.1038/s41598-018-22631-z. PubMed PMID: 29531263; PubMed Central PMCID: PMC5847565.	10.1038/s41598-018-22631-z
InflamNat	Drug-like	552	NA	Yes	Yes	No	Zhang R, Lin J, Zou Y, Zhang XJ, Xiao WL. Chemical Space and Biological Target Network of Anti-Inflammatory Natural Products. J Chem Inf Model. 2019 Jan 28;59(1):66–73. 10.1021/acs.jcim.8b00560. Epub 2018 Dec 20.	10.1021/acs.jcim.8b00560
Indofine Chemical Company Inc. natural products	Generalistic, industrial	56	No	Yes	No	No	x	x
InPACdb	Drug-like, plants, asia	124	No	Yes	Unknown	Unknown	Vetrivel U, Subramanian N, Pilla K. InPACdb--Indian plant anticancer compounds database. Bioinformation. 2009;4(2):71–74. Published 2009 Sep 5.	10.6026/97320630004071
InterBioScreen Ltd (IBS)	Generalistic, industrial	68,350	No	Yes	No	No	x	x
iSMART	tm, plants, asia	x	No	No	No	Yes	Kai-Wei Chang, Tsung-Ying Tsai, Kuan-Chung Chen, Shun-Chieh Yang, Hung-Jin Huang, Tung-Ti Chang, Mao-Feng Sun, Hsin-Yi Chen, Fuu-Jen Tsai & Calvin Yu-Chian Chen (2011) iSMART: An Integrated Cloud Computing Web Server for Traditional Chinese Medicine for Online Virtual Screening, de novo Evolution and Drug Design, Journal of Biomolecular Structure and Dynamics, 29:1, 243–25	10.1080/073911011010524988
KEGG	Metabolites	x	No	No	No	Yes	Minoru Kanehisa, Miho Furumichi, Mao Tanabe, Yoko Sato, Kanae Morishima, KEGG: new perspectives on genomes, pathways, diseases and drugs, Nucleic Acids Research, Volume 45, Issue D1, January 2017, Pages D353–D361	10.1093/nar/gkw1092
KNApSaCK	Plants	10,265	No	No	Yes	No	Kensuke Nakamura, Naoki Shimura, Yuuki Otabe, Aki Hirai-Morita, Yukiko Nakamura, Naoaki Ono, Md Altaf Ul-Amin, Shigehiko Kanaya, KNApSAcK-3D: A Three-Dimensional Structure Database of Plant Metabolites, Plant and Cell Physiology, Volume 54, Issue 2, February 2013, Page e4,	10.1093/pcp/pcs186
Lichen Database	Fungi	249	No	Yes	Yes	Yes	x	x
LOPAC1280 by Merck	Drug-like	1280	Unknown	Unknown	Unknown	Unknown	x	x
MAPS database	Plants, asia	x	No	Unknown	Unknown	Unknown	Ashfaq UA, Mumtaz A, Qamar TU, Fatima T. MAPS Database: Medicinal plant Activities, Phytochemical and Structural Database. Bioinformation. 2013;9(19):993–995. Published 2013 Dec 6.	10.6026/97320630009993
Marine Compound Database (MCDB)	Marine	182	No	Unknown	Unknown	Unknown	Babu PA, Puppala SS, Aswini SL, Vani MR, Kumar CN, Prasanna T. A database of natural products and chemical entities from marine habitat. Bioinformation. 2008;3(3):142–143.	10.6026/97320630003142
Marine Natural Product Database (MNPD)	Marine	6000	Unknown	Unknown	Unknown	Unknown	Lei J, Zhou J. A marine natural product database. J Chem Inf Comput Sci. 2002 May-Jun;42(3):742-8. PubMed PMID: 12086536.	10.1021/ci010111x
MarineLit	Marine	> 29,000	Yes	Yes	Unknown	Unknown	Blunt JW, Carroll AR, Copp BR, Davis RA, Keyzers RA, Prinsep MR. Marine natural products. Nat Prod Rep. 2018 Jan 16;35(1):8–53. Review. PubMed PMID: 29335692.	10.1039/c7np00052a
Massbank	Dereplication	x	No	No	No	Yes	Horai H, Arita M, Kanaya S, Nihei Y, Ikeda T, Suwa K, Ojima Y, Tanaka K, Tanaka S, Aoshima K, Oda Y, Kakazu Y, Kusano M, Tohge T, Matsuda F, Sawada Y, Hirai MY, Nakanishi H, Ikeda K, Akimoto N, Maoka T, Takahashi H, Ara T, Sakurai N, Suzuki H, Shibata D, Neumann S, Iida T, Tanaka K, Funatsu K, Matsuura F, Soga T, Taguchi R, Saito K, Nishioka T. MassBank: a public repository for sharing massspectral data for life sciences. J Mass Spectrom. 2010 Jul;45(7):703–14. 10.1002/jms.1777. PubMed PMID: 20623627.	10.1002/jms.1777
MedPServer	Plants, tm, asia, drug-like	1124	No	No	Yes	Yes	Potshangbam AM, Polavarapu R, Rathore RS, Naresh D, Prabhu NP, Potshangbam N, Kumar P, Vindal V. MedPServer: A database for identification of therapeutic targets and novel leads pertaining to natural products. Chem Biol Drug Des. 2019 Apr;93(4):438–446. 10.1111/cbdd.13430. Epub 2018 Nov 28. PubMed PMID:30381914.	10.1111/cbdd.13430
MetaCyc	Metabolites	x	No	Yes	Yes	Yes	Ron Caspi, Richard Billington, Carol A Fulcher, Ingrid M Keseler, Anamika Kothari, Markus Krummenacker, Mario Latendresse, Peter E Midford, Quang Ong, Wai Kit Ong, Suzanne Paley, Pallavi Subhraveti, Peter D Karp, The MetaCyc database of metabolic pathways and enzymes, Nucleic Acids Research, Volume 46, Issue D1, 4 January 2018, Pages D633–D639	10.1093/nar/gkx935
METLIN	Dereplication	x	No	No	No	Yes	Guijas C, Montenegro-Burke JR, Domingo-Almenara X, Palermo A, Warth B, Hermann G, Koellensperger G, Huan T, Uritboonthai W, Aisporna AE, Wolan DW, Spilker ME, Benton HP, Siuzdak G. METLIN: A Technology Platform for Identifying Knowns and Unknowns. Anal Chem. 2018 Mar 6;90(5):3156–3164. 10.1021/acs.analchem.7b04424. Epub 2018 Feb 9. PubMed PMID: 29381867; PubMed Central PMCID: PMC5933435.	10.1021/acs.analchem.7b04424
Mitishamba database	Plants, africa	1102	No	No	Yes	No	x	x
NADI	tm, plants	3000	Yes	Unknown	Unknown	Unknown	Ikram NK, Durrant JD, Muchtaridi M, Zalaludin AS, Purwitasari N, Mohamed N, Rahim AS, Lam CK, Normi YM, Rahman NA, Amaro RE, Wahab HA. A virtual screening approach for identifying plants with anti H5N1 neuraminidase activity. J Chem Inf Model. 2015 Feb 23;55(2):308–16. 10.1021/ci500405g. Epub 2015 Jan 29. PubMed PMID: 25555059; PubMed Central PMCID: PMC4340357.	10.1021/ci500405g
NANPDB	Plants, africa	6832	No	Yes	No	No	Ntie-Kang F, Telukunta KK, Döring K, Simoben CV, A Moumbock AF, Malange YI, Njume LE, Yong JN, Sippl W, Günther S. NANPDB: A Resource for Natural Products from Northern African Sources. J Nat Prod. 2017 Jul 28;80(7):2067–2076. 10.1021/acs.jnatprod.7b00283. Epub 2017 Jun 22. PubMed PMID: 28641017.	10.1021/acs.jnatprod.7b00283
NaprAlert	Generalistic	> 15,5000	Yes	Unknown	Yes	Unknown	Loub WD, Farnsworth NR, Soejarto DD, Quinn ML. NAPRALERT: computer handling of natural product research data. J Chem Inf Comput Sci. 1985 May;25(2):99–103. PubMed PMID: 4008538.	x
NAPROC-13	Dereplication	> 18,000	No	No	No	No (but has bibliographic references)	José Luis López-Pérez, Roberto Therón, Esther del Olmo, David Díaz, NAPROC-13: a database for the dereplication of natural product mixtures in bioassay-guided protocols, Bioinformatics, Volume 23, Issue 23, 1 December 2007, Pages 3256–3257	10.1093/bioinformatics/btm516
NCI DTP data	Drug-like	418	No	Yes	No	No	x	x
NeMedPlant	tm, plants, asia	100	No	No	Yes	No	Meetei PA, Singh P, Nongdam P, Prabhu NP, Rathore R, Vindal V. NeMedPlant: a database of therapeutic applications and chemical constituents of medicinal plants from north-east region of India. Bioinformation. 2012;8(4):209–211.	10.6026/97320630008209
NIST	Chemicals	x	Yes	Yes	Unknown	Unknown	x	x
NMRDATA	Dereplication	x	Unknown	Unknown	Unknown	Unknown	x	x
NMRShiftDB	Dereplication	1875	No	Yes	No	No	Kuhn S, Schlörer NE. Facilitating quality control for spectra assignments of small organic molecules: nmrshiftdb2--a free in-house NMR database with integrated LIMS for academic service laboratories. Magn Reson Chem. 2015 Aug;53(8):582–9. 10.1002/mrc.4263. Epub 2015 May 21. PubMed PMID: 25998807.	10.1002/mrc.4263
Novel Antibiotics database	Drug-like	5430	Yes	No	Yes	Unknown	x	x
NPACT	Plants, drug-like	1573	No	Yes	No	Yes	Manu Mangal, Parul Sagar, Harinder Singh, Gajendra P. S. Raghava, Subhash M. Agarwal, NPACT: Naturally Occurring Plant-based Anti-cancer Compound-Activity-Target database, Nucleic Acids Research, Volume 41, Issue D1, 1 January 2013, Pages D1124–D1129	10.1093/nar/gks1047
NPASS	Plants, bacteria, metazoa, fungi	30,858	No	Yes	Yes	Yes	Xian Zeng, Peng Zhang, Weidong He, Chu Qin, Shangying Chen, Lin Tao, Yali Wang, Ying Tan, Dan Gao, Bohua Wang, Zhe Chen, Weiping Chen, Yu Yang Jiang, Yu Zong Chen, NPASS: natural product activity and species source database for natural product research, discovery and tool development, Nucleic Acids Research, Volume 46, Issue D1, 4 January 2018, Pages D1217–D1222	10.1093/nar/gkx1026
NPAtlas	Bacteria, fungi	20,035	No	Yes	Yes	No	x	x
NPCARE	Plants, marine, bacteria, drug-like	1370	No	Yes	No but contains impact of nps on different cancer tissues and associated genes	Yes	Choi H, Cho SY, Pak HJ, et al. NPCARE: database of natural products and fractional extracts for cancer regulation. J Cheminform. 2017;9:2. Published 2017 Jan 5.	10.1186/s13321-016-0188-5
NPEdia	Generalistic	18,016	No	No	Yes	Yes	Takeshi Tomikia, Tamio Saitoa, Masashi Uekia, Hideaki Konnoa,Takeo Asaokab, Ryuichiro Suzukia, Masakazu Uramotoa, Hideaki, Kakeyaa, and Hiroyuki Osada. RIKEN Natural Products Encyclopedia (RIKEN NPEdia),a Chemical Database of RIKEN Natural Products Depository(RIKEN NPDepo). Journal of Computer Aided Chemistry , Vol.7, 157–162 (2006)	10.2751/jcac.7.157
NPL (library)	Plants, drug-like	814	NA	NA	Unknown	Unknown	Ronald J. Quinn, Anthony R. Carroll, Ngoc B. Pham, Paul Baron, Meredith E. Palframan, Lekha Suraweera, Gregory K. Pierens, Sorel Muresan. Developing a Drug-like Natural Product Library. J. Nat. Prod.2008713464-468 Publication Date:February 8, 2008	10.1021/np070526y
NuBBEDB	Plants, insects, america	2215	No	Yes	No	No	Pilon AC, Valli M, Dametto AC, et al. NuBBEDB: an updated database to uncover chemical and biological information from Brazilian biodiversity. Sci Rep. 2017;7(1):7215. Published 2017 Aug 3	10.1038/s41598-017-07451-x
Open Source Malaria	Drug-like	842	No	Yes	No	Yes	Williamson et al. Open Source Drug Discovery: Highly Potent Antimalarial Compounds Derived from the Tres Cantos Arylpyrroles. ACS Cent Sci. 2016 Oct 26;2(10):687–701. Epub 2016 Sep 14.	10.1021/acscentsci.6b00086
p-ANAPL (Pan-African Natural Product Library )	Plants, africa	538	No	NA	No	No	Ntie-Kang F, Amoa Onguéné P, Fotso GW, et al. Virtualizing the p-ANAPL library: a step towards drug discovery from African medicinal plants. PLoS One. 2014;9(3):e90655. Published 2014 Mar 5.	10.1371/journal.pone.0090655
PAMDB	Metabolites, bacteria	x	No	Yes	Yes	Yes	Weiliang Huang, Luke K Brewer, Jace W Jones, Angela T Nguyen, Ana Marcu, David S Wishart, Amanda G Oglesby-Sherrouse, Maureen A Kane, Angela Wilks, PAMDB: a comprehensive Pseudomonas aeruginosa metabolome database, Nucleic Acids Research, Volume 46, Issue D1, 4 January 2018, Pages D575–D580	10.1093/nar/gkx1061
Phenol-explorer	Food	862	No	Yes	Yes	Yes	Joseph A. Rothwell, Jara Perez-Jimenez, Vanessa Neveu, Alexander Medina-Remón, Nouha M’Hiri, Paula García-Lobato, Claudine Manach, Craig Knox, Roman Eisner, David S. Wishart, Augustin Scalbert, Phenol-Explorer 3.0: a major update of the Phenol-Explorer database to incorporate data on the effects of food processing on polyphenol content, Database, Volume 2013, 2013, bat070	10.1093/database/bat070
Phytochemica	Plants, tm, asia	571	No	No	Yes	Supposedly	Pathania,S., Ramakrishnan,S.M., and Bagler,G. Phytochemica: a platform to explore phytochemicals of medicinal plants. Database (2015) Vol. 2015: article ID bav075;	10.1093/database/bav075
PhytoHub	Food, plants	1200	No	No	Yes	Yes	x	x
Pi Chemicals System Natural Products	Generalistic, industrial	405	No	No	No	No	x	x
Prestwick	Plants, industrial	320	Yes	Unknwn	Unknown	Unknown	x	x
ProCarDB	Structure, bacteria	304	No	No	Yes	Yes	Nupur, Vats A, Dhanda SK, Raghava GPS, Pinnaka A, Kumar A (2016):”ProCarDB: a database of bacterial carotenoids”; BMC Microbiology 16(96)	10.1186/s12866-016-0715-6
PubChem	Chemicals	3529	No	Yes	No	Yes	Sunghwan Kim, Paul A. Thiessen, Evan E. Bolton, Jie Chen, Gang Fu, Asta Gindulyte, Lianyi Han, Jane He, Siqian He, Benjamin A. Shoemaker, Jiyao Wang, Bo Yu, Jian Zhang, Stephen H. Bryant, PubChem Substance and Compound databases, Nucleic Acids Research, Volume 44, Issue D1, 4 January 2016, Pages D1202–D1213	10.1093/nar/gkv951
REAXYS	Chemicals	> 220,000	Yes	Unknown	Unknown	Unknown	x	x
ReSpect	Dereplication	4767	No	Yes	Yes	Yes	Sawada Y, Nakabayashi R, Yamada Y, Suzuki M, Sato M, Sakata A, Akiyama K, Sakurai T, Matsuda F, Aoki T, Hirai MY, Saito K. RIKEN tandem mass spectral database (ReSpect) for phytochemicals: a plant-specific MS/MS-based data resource and database. Phytochemistry. 2012 Oct;82:38–45. 10.1016/j.phytochem.2012.07.007. Epub 2012 Aug 4. PubMed PMID: 22867903	10.1016/j.phytochem.2012.07.007
SANCDB	Plants, africa	623	No	No	Yes	Yes	Hatherley R, Brown DK, Musyoka TM, Penkler DL, Faya N, Lobb KA, Tastan Bishop Ö. SANCDB: a South African natural compound database. J Cheminform. 2015 Jun 19;7:29. 10.1186/s13321-015-0080-8. eCollection 2015. PubMed PMID: 26097510; PubMed Central PMCID: PMC4471313	10.1186/s13321-015-0080-8
Seaweed Metabolite Database (SWMD)	Marine	1110	No	Yes	Yes	No	Davis GD, Vasanthi AH. Seaweed metabolite database (SWMD): A database of natural compounds from marine algae. Bioinformation. 2011;5(8):361–364. Published 2011 Jan 22.	10.6026/97320630005361
Specs Natural Products	Generalistic, industrial	745	No	Unknwon	No	No	x	x
Spektraris NMR	Dereplication	248	No	Yes	No	Yes	Fischedick JT, Johnson SR, Ketchum RE, Croteau RB, Lange BM. NMR spectroscopic search module for Spektraris, an online resource for plant natural product identification--Taxane diterpenoids from Taxus × media cell suspension cultures as a case study. Phytochemistry. 2015 May;113:87–95. 10.1016/j.phytochem.2014.11.020. Epub 2014 Dec 19. PubMed PMID: 25534952; PubMed Central PMCID: PMC4441555	10.1016/j.phytochem.2014.11.020
StreptomeDB	Bacteria	6415	No	No	Yes	No	Dennis Klementz, Kersten Döring, Xavier Lucas, Kiran K. Telukunta, Anika Erxleben, Denise Deubel, Astrid Erber, Irene Santillana, Oliver S. Thomas, Andreas Bechthold, Stefan Günther, StreptomeDB 2.0—an extended resource of natural products produced by streptomycetes, Nucleic Acids Research, Volume 44, Issue D1, 4 January 2016, Pages D509–D514	10.1093/nar/gkv1319
Super Natural II	Generalistic	320,670	No	No	No	To supplyers only	Priyanka Banerjee, Jevgeni Erehman, Björn-Oliver Gohlke, Thomas Wilhelm, Robert Preissner, Mathias Dunkel, Super Natural II—a database of natural products, Nucleic Acids Research, Volume 43, Issue D1, 28 January 2015, Pages D935–D939	10.1093/nar/gku886
Super Scent	Other	2100	No	No	No	Yes	Mathias Dunkel, Ulrike Schmidt, Swantje Struck, Lena Berger, Bjoern Gruening, Julia Hossbach, Ines S. Jaeger, Uta Effmert, Birgit Piechulla, Roger Eriksson, Jette Knudsen, Robert Preissner, SuperScent—a database of flavors and scents, Nucleic Acids Research, Volume 37, Issue suppl_1, 1 January 2009, Pages D291–D294	10.1093/nar/gkn695
Super Sweet	Food, metabolites	15,000	No	No	No	Yes	Jessica Ahmed, Saskia Preissner, Mathias Dunkel, Catherine L. Worth, Andreas Eckert, Robert Preissner, SuperSweet—a resource on natural and artificial sweetening agents, Nucleic Acids Research, Volume 39, Issue suppl_1, 1 January 2011, Pages D377–D382	10.1093/nar/gkq917
TargetMol Natural Compound Library	Generalistic, industrial	1680	Yes	No	Unkown	Unknown	x	x
TC-MC	tm, asia, plants	> 20,000	No	No	Yes	Yes	Kim SK, Nam S, Jang H, Kim A, Lee JJ. TM-MC: a database of medicinal materials and chemical compounds in Northeast Asian traditional medicine. BMC Complement Altern Med. 2015 Jul 9;15:218. 10.1186/s12906-015-0758-5. PubMed PMID: 26156871; PubMed Central PMCID: PMC4495939.	10.1186/s12906-015-0758-5
TCMDB@Taiwan	tm, asia, plants	58,351	No	Yes	No	No	Chen CY. TCM Database@Taiwan: the world’s largest traditional Chinese medicine database for drug screening in silico. PLoS One. 2011 Jan 6;6(1):e15939. 10.1371/journal.pone.0015939. PubMed PMID: 21253603; PubMed Central PMCID: PMC3017089	10.1371/journal.pone.0015939
TCMID	tm, asia, plants	12,549	No	No	Yes (but difficutl to extract)	Yes	10.1093/nar/gks1100Xue R, Fang Z, Zhang M, Yi Z, Wen C, Shi T. TCMID: Traditional Chinese Medicine integrative database for herb molecular mechanism analysis. Nucleic Acids Res. 2013 Jan;41(Database issue):D1089–95. 10.1093/nar/gks1100. Epub 2012 Nov 29. PubMed PMID: 23203875; PubMed Central PMCID: PMC3531123.	10.1093/nar/gks1100
TCMSP	tm, asia, plants	29,384	No	Unknown	Unknown	Unknown	Ru J, Li P, Wang J, et al. TCMSP: a database of systems pharmacology for drug discovery from herbal medicines. J Cheminform. 2014;6(1):13. Published 2014 Apr 16.	10.1186/1758-2946-6-13
TIM	tm, asia, plants	1829	No	No	Unknown	Unknown	Polur H, Joshi T, Workman CT, Lavekar G, Kouskoumvekaki I. Back to the Roots: Prediction of Biologically Active Natural Products from Ayurveda Traditional Medicine. Mol Inform. 2011 Mar 14;30(2–3):181–7. 10.1002/minf.201000163. Epub 2011 Feb 10	10.1002/minf.201000163
TIPdb	Asia, plants, drug-like	8656	No	Yes	No	No	Chun-Wei Tung, Ying-Chi Lin, Hsun-Shuo Chang, Chia-Chi Wang, Ih-Sheng Chen, Jhao-Liang Jheng, Jih-Heng Li, TIPdb-3D: the three-dimensional structure database of phytochemicals from Taiwan indigenous plants, Database, Volume 2014, 2014, bau055	10.1093/database/bau055
TMDB	Plants, metabolites	1393	No	Unknown	Yes	Yes	Yue Y, Chu GX, Liu XS, et al. TMDB: a literature-curated database for small molecular compounds found from tea. BMC Plant Biol. 2014;14:243. Published 2014 Sep 16. 10.1186/s12870-014-0243-1	10.1186/s12870-014-0243-1
TPPT	Plants, toxins, europe	1583	No	Yes	Yes	Yes	Günthardt BF, Hollender J, Hungerbühler K, Scheringer M, Bucheli TD. Comprehensive Toxic Plants-Phytotoxins Database and Its Application in Assessing Aquatic Micropollution Potential. J Agric Food Chem. 2018 Jul 25;66(29):7577–7588. 10.1021/acs.jafc.8b01639. Epub 2018 Jul 16. PubMed PMID: 29944838.	10.1021/acs.jafc.8b01639
TriForC	Plants	266	No	No	Yes	Yes	Karel Miettinen, Sabrina Iñigo, Lukasz Kreft, Jacob Pollier, Christof De Bo, Alexander Botzki, Frederik Coppens, Søren Bak, Alain Goossens, The TriForC database: a comprehensive up-to-date resource of plant triterpene biosynthesis, Nucleic Acids Research, Volume 46, Issue D1, 4 January 2018, Pages D586–D594	10.1093/nar/gkx925
UEFS	Plants, america	503	No	Yes	No	No	x	x
UNPD (Universal Natural Products Database)	Generalistic	213,100	No	Yes	No	No	Gu J, Gui Y, Chen L, Yuan G, Lu HZ, Xu X. Use of natural products as chemical library for drug discovery and network pharmacology. PLoS One. 2013 Apr 25;8(4):e62839. 10.1371/journal.pone.0062839. Print 2013. PubMed PMID: 23638153; PubMed Central PMCID: PMC3636197	10.1371/journal.pone.0062839
VIETHERB	Plants, asia	10,887	No	Unknown	Unknown	Unknown	Nguyen-Vo TH, Le T, Pham D, Nguyen T, Le P, Nguyen A, Nguyen T, Nguyen TN, Nguyen V, Do H, Trinh K, Duong HT, Le L. VIETHERB: A Database for Vietnamese Herbal Species. J Chem Inf Model. 2019 Jan 28;59(1):1–9. 10.1021/acs.jcim.8b00399. Epub 2018 Dec 3. PubMed PMID: 30407009.	10.1021/acs.jcim.8b00399
Yeast Metabolome Database	Metabolites, dereplication	16,042	No	Yes	Yes	Yes	Ramirez-Gaona M, Marcu A, Pon A, et al. YMDB 2.0: a significantly expanded version of the yeast metabolome database. Nucleic Acids Res. 2017;45(D1):D440–D445. 10.1093/nar/gkw1058	10.1093/nar/gkw1058
YaTCM	tm, asia, plants	47,696	No	No	No	No	Li B, Ma C, Zhao X, Hu Z, Du T, Xu X, Wang Z, Lin J. YaTCM: Yet another Traditional Chinese Medicine Database for Drug Discovery. Comput Struct Biotechnol J. 2018 Nov 23;16:600–610. 10.1016/j.csbj.2018.11.002. eCollection 2018. PubMed PMID: 30546860; PubMed Central PMCID: PMC6280608.	10.1016/j.csbj.2018.11.002
ZINC natural products catalogue	Generalistic	85,198	No	Yes	No	No	Sterling T, Irwin JJ. ZINC 15--Ligand Discovery for Everyone. J Chem Inf Model. 2015;55(11):2324–2337. 10.1021/acs.jcim.5b00559	10.1021/acs.jcim.5b00559

The databases are sorted by alphabetical order of their names and the table lists their various features such as: if they are open or commercial, if they are maintained and updated, what type of NPs they contain and their origin, the approximative number of molecular structures they contain, most recent publication of the collection, if a registration is required to access the data, if extensive metadata is available (taxonomy of the organism producing the NP, tissue, the geographical location where it is isolated, it’s application in (traditional) medicine, diseases it targets, etc.) and if the download of the molecular structures for local use (such as virtual screening) is easy. All these criteria are chosen to evaluate the “FAIRness” [23] (Findable, Accessible, Interoperable and Reusable) of the NP resources.

For the purpose of this review, the first classification level of the NP databases is their open or commercial access. Next, among the open-access databases, we distinguish databases of metabolites (that contain NPs but also products of primary metabolism), generalistic databases, that do not limit themselves to a particular geographic location or taxonomic classification, databases containing experimental spectra of NPs (NMR, mass spectrometry) and can be used for dereplication applications, thematic databases, that focus on traditional medicine, on drug-like NPs, on the biodiversity of a particular geographic region or on a particular taxonomic group and, finally, open-access industrial catalogues, that are virtual collections of NPs that chemical companies synthesize or isolate and sell. Of course, this segregation is not the only one possible and was made here uniquely for the readability purpose.

### Commercial databases

Commercial databases sell the data, access or licence, and in general, it is quite expensive [24], even for academic use (from 6600 US$ per year for the Dictionary of Natural Products [25] to over 40,000 US$ for Reaxys [26] and SciFinder [27]).

The Chemical Abstracts Service (CAS) launched in 1995 SciFinder [27], a curated database of chemical information, compiled and maintained by the American Chemical Society. Originally available as desktop software, the web version of SciFinder is available since 2008. As it is CAS that assigns a unique registry number to every chemical substance described in the scientific literature since 1957, the SciFinder contains one, if not the biggest collection of curated chemicals, and, subsequently, of NPs. It is estimated that the number of NPs in SciFinder is over 300,000.

Reaxys [26] is a database for substances, reactions and documents compiled and maintained by the editor Elsevier. It contains over 10^7^ compounds in total, over 200,000 of which are NPs.

The Dictionary of Natural products (DNP) [25] and it’s autonomous sub-sections, the Dictionary of Marine Natural Products (DNMP) [28] and the Dictionary of Food Compounds [29], are the considered as the most complete and best-curated resources for NP.

NaprAlert [30] was created by researchers at the University of Chicago and contains manually curated information on NPs from literature with rich metadata. Nowadays offers limited free searchers under conditions for academic researchers.

National Institute of Standards and Technology-NIST (version 17) [31] is one of the standard reference databases for mass spectra (MS) data and is developed and maintained at the National Institute of Health (NIH) in the USA. The main library contains over 250,000 molecules of natural origin (the separation between primary metabolites and NPs is not clearly marked) and is only purchasable on a compact disk.

MarinLit [32, 33] is a database of marine NPs based on literature reviews and contain highly curated data that has been collected since the 1970s at the University of Canterbury, New Zealand, and since several years is maintained by the Royal Society of Chemistry (RSC). AntiMarin [34, 35] is a historic database of marine NPs that have a described antibiotic activity. While it is still widely cited in thematic studies, the database itself is not accessible anymore, as was apparently merged with MarinLit.

AntiBase [36] is a comprehensive database of more than 40,000 NPs from microorganisms and higher fungi with very rich metadata collected from literature and manually validated. It is not updated since 2014 and is only available for purchase on Wiley’s website [37].

eBasis (Bioactive Substances in Food Information Systems) is an online, manually curated collection of 267 foods and 794 active compounds that they contain. The database offers rich and high-quality metadata on food NP activities and structures and limited free access to scientists to try the resource.

The Natural Product Discovery System (NADI) [38] contains over 3000 natural compounds from more than 15,000 Malaysian plant species. Despite being developed and maintained by the University Sains Malaysia, it is not open for academic use.

ChemTCM [39] is a database of NPs from plants used in traditional Chinese herbal medicine. The original part of this dataset resides not only in the very rich metadata but also in the predicted activity of NPs against common Western therapeutic targets and their estimated molecular activity according to traditional Chinese herbal medicine categories. The database was developed at King’s College London, in the UK, in part with the support of Innovation China-UK.

The Natural Products Library (NPL) [40] was described in a paper by AstraZeneca, a famous pharmaceutical company, but the data, containing at the moment of publication over 800 well-curated and annotated NPs, only remained as an in-house collection.

The Ayurveda dataset [41] was initially a published database of NPs extracted from the Indian traditional medicine plants. The link in the mentioned publication is still working but redirects to a website that provides software solutions for NP and chemistry research in general. Maybe the database is still available together with the software, but the access to it is for subscriptions only.

The Berdy’s Bioactive Natural Products Database [42] database is mentioned in publications from the 2000s and early beginning of 2010s but is not accessible anymore not even for the purchase of an older version. Originally, Birdy’s company was sending the database as a paper version and with the rise of accessible digital storage, on a digital medium upon order. The company does not seem to exist anymore.

### Open-access databases

We could identify a total of 92 open-access NP resources across the literature in the last 20 years. The concept of “Open-access” encourages and prioritizes free and open online access to academic information, such as data and scientific publications. For a dataset, whether in a database or attached as additional information to an article, it means that anyone can read, download, copy, distribute, print, search for and within and re-use all or parts of data that are contained in it. For this review, we have endeavoured to compile an exhaustive list of open-access NP resources that have been cited at least ones in a peer-reviewed scientific publication after the year 2000. As the number of such sources is quite substantial (87), a thematic classification for them has been established. First, we present larger databases of organic molecules that also contain metabolites and NPs. These are followed by the presentation of databases containing molecular spectra (mass spectrometry or NMR) that can be used for the dereplication process for the identification of organic molecules and, in particular, of NPs in experimental data. Next, the scope will be narrowed with databases containing only NPs but without any taxonomic, usage or geographic selection on them. The most diverse data source category is the so-called “thematic” one: it contains databases of NPs that focus on a particular taxonomy (e.g. plants, bacteria, fungi), on a particular usage (e.g. Chinese, Indian or African traditional medicine, NPs found in food or toxic NPs) or on a particular geographic location (e.g. marine NPs, Brazilian and Mexican biodiversity NPs). Finally, are introduced industrial catalogues of NPs. These are made available by chemical companies that synthesize or purify NPs on command.

### Databases of metabolites and chemicals

The first starting points in the search for structures for organic molecules are these big chemical libraries. They contain a wide range of organic compounds, and metabolites and NPs are well identifiable in them. The reference libraries, widely accepted by the scientific community as sources of reliable molecular information are: ChEBI [43], ChEMBL [44], ChemSpider [45], PubChem [46] and ChemBank [47]. ChEBI is developed and maintained at the European Bioinformatics Institute (EBI) and its main focus is chemical ontologies, i.e. structural relationships between molecules; it contains over 15,000 clearly identified NPs. ChEMBL is also the product of EBI but it has a wider focus and is considered as a repository for experimentally elucidated molecular structures and, in particular, drugs and drug-like chemical; it contains over 1800 NPs, but this number is very probably underestimated because of the unclear labelling of molecules as NP in this database. PubChem is an integrated platform of small molecules and biological activities is an initiative of the US (NIH) and is one of the major sources for biomolecules discovery and submission. It contains over 3500 NPs, although, similarly to ChEMBL, this number is very underestimated due to the unclear labelling of compounds as NPs. ChemSpider is a chemical database offering very rich metadata, cross-references to a lot of other chemical sources and advanced search. It is maintained by the Royal Society of Chemistry and contains over 9700 easily findable NPs. ChemBank was developed by the Broad Institute of Harvard and MIT and was dedicated to the storage of raw screening data of small organic molecules. This resource is unfortunately not available anymore due to maintenance difficulties, although all data remains available for a bulk download, but is not as handy to search.

There are also databases that focus only on metabolites, chemicals that are produced by living organisms (generally, but not only through enzyme-catalyzed reactions) and that are involved in primary and secondary metabolisms. The two major and most comprehensive databases for metabolites covering most of the domains of life are KEGG [48] and MetaCyc [49]. They contain an equivalent amount of chemicals, also involved in secondary metabolism, i.e. NPs, but present a different point of view on data organization and have been widely compared in the literature [50]. The BRENDA database [51] focuses on enzyme activities, but also contains the compounds involved in enzyme-catalyzed reactions, and this, covering most of all known domains of life. The particularity of this database is the manually validated compounds, reactions and enzyme activities in its main part, and exhaustive taxonomic origins for enzymes and compounds; however, NPs and primary metabolites are not clearly separated in this resource, so it is difficult to estimate their respective numbers. The Chemical Structure Lookup Service (CSLS) [52] was developed for a very rapid metabolite structure lookup in an aggregated collection of more than 80 databases comprising more than 27 million unique structures in 2007. Not updated anymore, it is still possible to download the datasets, but the lookup service is not available so the extraction of NPs only requires an extensive data curation. The last database presented in this section is BiGG [53]: a platform for highly-curated genome-scale metabolic models. It contains, as parts of the metabolic models metabolites, but the distinction of primary and secondary metabolism is not clear, so it requires a lot of efforts to extract information on NPs only.

### Databases for dereplication

Dereplication is one important step in experimental NP discovery as it prevents re-isolation and re-characterization of already known molecules. It consists of a lookup in databases with annotated experimental data (mainly mass spectrometry (MS) and Nuclear Magnetic Resonance (NMR) spectra) for comparison to newly obtained experimental data, and its annotation in case of found spectral identity. There are two big categories of databases used for dereplication based on the type of spectra they contain, MS and NMR.

#### Databases for dereplication for MS data

There are three distinct databases called “MassBank”: the MassBank of North America (MoNa) [54], the European MassBank [55] and the Japanese MSSJ MassBank [56]. The three contain reference MS spectra for metabolites and extensive metadata. MoNa tends to be favoured by the scientific community as it integrates data from more sources than the two others, contains rich and community-curated metadata and facilitates the submission of new datasets.

METLIN [57] is a database that allows the characterization of known metabolites and a technology platform for the identification of known and unknown metabolites and other chemical entities. It is a comprehensive resource containing over 1 million molecules including primary metabolites, toxins, small peptides, and NPs. METLIN’s high-resolution tandem mass spectrometry (MS/MS) database, which plays a key role in the identification process, has data generated from both reference standards and their labelled stable isotope analogues, facilitated by METLIN-guided analysis of isotope-labelled microorganisms. However, it does not allow an easy download of the data, but the access to the platform is free for academic use.

The Human Metabolome Database (HMDB) [58] is a metabolomic database containing comprehensive information on human metabolites with very extensive metadata and reference spectra. It contains human-produced NPs together with NPs that are essential for the function of the human organism. However, as it is the case in a lot of previously described databases, the separation between NPs and primary metabolites is tricky.

From the same institution, the Yeast Metabolome Database (YMDB) [59], was created with the same pattern as the HMDB, and therefore also contains very extensive metadata for baker’s yeast metabolites, enzymes that are involved in the molecular metabolism and reference spectra. Again, the separation between NPs and primary metabolites is difficult, do this dataset was not included in further analysis either.

The RIKEN MSn spectral database for phytochemicals (ReSpect) is a collection of in-house and literature MS plant NP spectra. The website is still maintained and is usable but the last dataset has been added in 2013.

The Global Natural Products Social Molecular Networking (GNPS) [60] is a web-based knowledge base containing MS spectra for NPs only and is intended to be the base for the community-wide organization and sharing of raw, processed or identified data. In addition to providing access to spectra, it is also possible to download solely the structures of the NPs from this database.

#### Databases for dereplication for NMR data

NMRshiftDB [61] an open and peer-reviewed database for organic molecules structures and their NMR spectra. It contains a big number of easily identifiable NP spectra that makes it the reference tool for NP dereplication applications.

NMRdata [62] is a Chinese initiative for the storage and elucidation of NP structures from NMR data. Unfortunately, the main website is in Chinese and the English version is limited. To access the data one needs an account in a university that participates in the NMRdata project. At the moment of the writing of this manuscript, NMRdata contains 1,167,468 spectra, which theoretically makes it the biggest resource for NMR data in the world but it is under-used due to the language barrier.

NAPROC-13 [63] is a database containing 13C spectral information of over 6000 natural compounds. All data is accessible and searchable online, however, it is not possible to download the subsequent structures.

Spektraris NMR database [64] is a collection of NMR spectra that are focusing on plant NPs. The more than 400 spectra from more than 200 compounds in this database were manually transcribed from the literature. Spectra from this database are also submitted to NMRshiftDB to profit of the advanced technological aspects of the latter.

### Generalistic databases of natural products

Generalistic public databases for NPs are not specialized in any particular type of NP nor on NP origins or usages. They are generally intended as catalogues for various purposes, such as in silico screening for activity prediction, molecular docking and so on. Seven generalistic public NP databases that have been active in the last 20 years have been identified from the literature.

SuperNatural II [65] is a database that contains over 300,000 NPs together with their 2D structures, computed physicochemical properties and predicted toxicity. It also provides references to the chemical suppliers for the actual purchase of the molecules, but not to other chemical databases. The database is maintained but is probably not updated anymore as some of the companies selling molecules are not active anymore (such as MDPI [66]). Unfortunately, SuperNatural does not provide a bulk download, even if the download of separate MOL files for molecules is possible and erroneously does not contain only NPs (e.g. it contains dodecahedrane, identified in this database under SN00136231 and it is not a NP), so this resource needs to be used with caution despite its wide fame in the scientific community.

The Universal Natural Products Database (UNPD) [67] was an effort to compile all know NPs in one collection for in silico drug screening. The last accessible version of the UNPD contains over 200,000 NP structures. The database is not accessible anymore through the link provided in the original publication, but a copy of the molecular structures contained in it is still maintained on the ISDB [68] website (a database for in silico predicted MS/MS spectra for NPs).

ZINC [69] is a public access database and toolset that was initially developed to enable easy access to chemical compounds for virtual screening purposes and that became ever widely used for a big range of cheminformatic applications. It has a very clear separation of molecules in catalogues, in particular on their origin, and contains an easily searchable and retrievable collection of over 85,000 NPs.

The Natural Product Activity and Species Source Database (NPASS) [70] contains over 30,000 NPs from plants, bacteria, fungi and animals and is developed and maintained at the National University of Singapore. This database was created to provide a reliable source for highly curated NPs with structures, experimental activity values and the organisms that synthesize them.

RIKEN Natural Products Encyclopedia (NPEdia) [71] contains over 25,000 secondary metabolites isolated from various species and annotated with rich metadata, such as molecule origin and physicochemical and biological properties. The database is still available online but is not updated since 2014.

3DMET [72] is a database that was created in 2005 in the National Institute of Agrobiological Sciences in Japan and is still maintained and updated until now. The idea of such a database came during the conversion from 2D to 3D NP structures and the errors that were occurring during it that needed manual curation. Currently, the database contains over 18,000 entries, cross-referenced to the KEGG database [48], but unfortunately, the download of the structures is not possible.

The Chinese Natural Products Database (CNPD**)** [73] is a generalistic database created by Chinese researchers in order to facilitate the virtual screening of NPs for drug discovery purposes. This database is mentioned in over 120 papers until 2010 but is impossible to localize, as there is no URL provided in the original publication of the database and the dataset is not added as supplementary information to it. It is therefore probably incorrect to cite this database as a data source for NP, as the only possible sources found (from NeoTrident Technology Ltd) are in Chinese only.

One big negative point is that in ZINC, SuperNatural II and UNPD databases, the three biggest ones in terms of the number of NPs, the taxonomic nor geographic origins of the organism that produced the compound cannot be identified and in general they lack metadata and literature references.

For the completeness of this list, it is also necessary to site two major tools for the discovery and prediction of NPs from protein sequence data: antiSMASH [74] and PRISM [75]. Both are trained on, among others, NP data, but the latter is not provided directly to the public.

### Thematic databases

Thematic databases for NPs focus on one particular origin or application of these secondary metabolites. Here we list databases that contain NPs produced by a particular domain of life (e.g. plants, fungi, bacteria), produced by organisms living in a particular geographical location (e.g. marine organisms, South American organisms) or by its application (traditional medicines, food or drugs). Apart from some rare exceptions, thematic databases tend to be small (less than 3000 entries) and very specialized.

In order to avoid biological provenance confusion, it needs to be noted that in some cases, NPs isolated from plants and animals can actually be synthesized by microorganisms that live on or in the host [76]. This is particularly the case of endophytes, bacteria living inside plant cells and very difficult to differentiate from the latter during preparation for metabolomics experiments [77]. Although the confusion is rare due to the improvement of identification methods and genetic approaches, it can create a bias in reproducibility of the NP isolation and needs, therefore, to be taken into account.

#### Natural products by the taxonomy of the synthesizing organism

##### Plants

KNApSaCK [78] is a comprehensive database for plant NPs that contains over 10,000 retrievable 2D and 3D structures, information on the relationships between the NPs and their expressing organism(s). It is pretty difficult to navigate despite the original design choices, and it does not offer a bulk download of the dataset.

Collective Molecular Activities of Useful Plants (CMAUP) [79], a relatively new database, contains very extensive information on plants that are linked to human activities together with their chemical constituents, i.e. NPs. The database offers very rich metadata for NPs, such as the plants that produce them and their geographical distributions.

TriForC [80] is a European Union-funded project that aims for the “discovery and production of known and novel bioactive triterpenes for pharmaceutical and agrochemical development”. The database contains a pipeline for triterpenes discovery and 266 NPs together with the enzymes and pathways leading to their production. It contains metadata for the compounds, but no structures in computer-readable format nor the possibility of downloading them.

Alkamid database [81] references over 300 *N*-alkylamides from plants, a promising group of bioactive compounds in drug and crops research. The database is fully open and offers rich metadata, in particular, the taxonomical classification of plants that produces the NPs, but does not allow a bulk download of any information from it.

The Tea Metabolome Database (TMDB) [5] is a curated and literature-based database for tea components. Not accessible anymore, it contained over 1300 constituents found in tea.

##### Microorganisms

StreptomeDB [82] is a collection of NPs from bacteria from the Streptomyces genus, which is very important for the production of natural bioactive compounds such as antibiotics, antitumour and immunosuppressant drugs. These bacteria are of particular importance in pharmacological research as around two-thirds of all known natural antibiotics are produced by them. While collecting data for this review, we encountered some difficulties to access the website, but the data was downloadable. In addition, an old dataset is available on ZINC.

The Natural Products Atlas (NP Atlas) [83] is maintained at the Simon Fraser University in Canada and is curated by a consortium of data curators around the world. It is designed to cover NPs from microbes (bacteria, fungi, lichens and cyanobacteria) published in the peer-reviewed literature. The resource is actively updated, allows a bulk download of all data and metadata and since September 2019 is completely open.

ProCarDB [84] is a database for carotenoids produced by bacteria. It contains over 300 compounds with rich metadata and structures but does not offer any download option.

PAMDB [85] is a comprehensive Pseudomonas aeruginosa metabolome database, well-curated, with rich metadata and offering bulk download. However, it does not contain only NPs but also results of the primary metabolism, so it was not included in the COCONUT collection.

The Lichen Database [86] is a collection of over 200 metabolites that have been isolated and identified experimentally in lichens. The database is not available yet, but the data has been already published in the MetaboLights [87] repository for metabolomics experimental data.

#### Natural products by use

##### Traditional medicines

The World Health Organization listed between 1999 and 2009 a list of over 21 000 plants used for medicinal purposes all over the world [88, 89]. This effort was made for proper identification of safe plants, as it is estimated that plant-based traditional medicines are used by 60% of the world’s population [90]. In addition to efforts to establish formal, DNA-based identification of such plants for wider use [91], collections of medicinal plant species, and in particular of phytochemicals, NPs produced by plants, associated to their therapeutic activities and physicochemical properties are being established around the world. This is particularly the case in Asia and Africa, where traditional medicines remain an important part of everyday life for cultural, traditional and economic reasons.

Traditional Chinese Medicine (TCM) is naturally part of the Chinese public health system [92, 93]. It is therefore coherent that in this country the scientific study of natural compounds from plants used in TCM is very advanced and is receiving strong governmental support, and they have developed a plethora of databases containing NPs, their sources and effects.

The biggest database containing NPs used in TCM is TCM@Taiwan [94]. It contains over 58,000 entries and is directly feeding iSMART [95], an integrated cloud computing web server for online virtual screening, evolution studies and drug design. In addition to this, there are several other, smaller, databases for NPs TCM that can be cited, such as the Chinese Ethnic Minority Traditional Drug Database (CEMTDD) [96], that is maintained, but not updated and contains 4000 NPs, the Chinese Traditional Medicinal Herbs Database (CHDD) [97], not maintained anymore, but according to the publication contained over 30,000 entries, now not accessible and probably lost for the scientific community. Some other databases containing phytochemicals and other active compounds used in TCM can be cited, such as the Comprehensive Herbal Medicine Information System for Cancer (CHMIS-C) [98] that is not maintained anymore, the Encyclopaedia of Traditional Chinese Medicine (ETCM) [99], that is maintained but the chemical structures it contains are not easily retrievable, the database of medicinal materials and chemical compounds in Northeast Asian TM (TM-MC) [100], which is maintained, updated, but no structures but contains precise plant species for all compounds, the Traditional Chinese Medicine Integrative Database (TCMID) [101], maintained, but not updated anymore, The Traditional Chinese Medicine Systems Pharmacology database and analysis platform (TCMSP) [102], that is also not maintained anymore but used to contain over 29,000 NPs. One can quickly realize that there is a lot of databases that focus on chemical compounds used in TCM, and creators of the latter recognize it: there is even a database called “Yet Another Traditional Chinese Medicine Database” (YaTCM) [103] that was published in 2018. Mainly, all these databases differ in the number of compounds they cover, in the richness of their metadata and on the availability of the datasets they contain.

Another extremely important traditional medicine in Asia is the Indian Ayurveda, that also got a wide popularization worldwide over the past decade. There are, however, very few databases listing natural compounds from plants, insects and animals used in Ayurveda, and they do not contain as many entries as the Chinese ones. Only two are currently online and open. The first one, IMPPAT [104] is the manually curated database of over 10,000 phytochemicals extracted from 1700 Indian medicinal plants, their phytochemistry and their therapeutic effects. The other, MedPServer [105] contains NPs from plants from North-East India used in traditional medicine. It aims towards the understanding of the therapeutic mechanisms of action of the 1124 NPs from these plants by integrating ligand-based and structure-based approaches. NeMedPlant [106] is a small (over 100 NPs) database of active compounds from plants used in North-East Indian traditional medicine, with rich metadata focused on the plants that produce the compound but without possibilities of downloading any information and is not updated anymore. Because it was cited in several peer-reviewed papers, we also need to mention TIM [90], the database created in 2011 for the Prediction of Biologically Active Natural Products from Ayurveda Traditional Medicine but never linked to an actual database not listing the NPs in the supplementary material of the publication.

Phytochemica [107] is a small database of plant-derived chemicals that contains plants from Himalaya used in both Chinese and Indian traditional medicines. There are also some databases of NPs that specialize in traditional medicines of other parts of Asia, such as the Database of Indonesian Medicinal Plants [108] and TIPdb [109] for plants from Taiwan, but most of them are relatively small and contain in general only few hundreds of compounds.

African Traditional Medicine (ATM) is the other extremely rich and developed traditional medicine with a lot of modern efforts to study, rationalize and put its teachings to the benefit of modern medicine. As for the CTM and the Ayurveda, it requires inventorying plants used by African traditional doctors, identifying the parts that are used to efficiently cure and then identify the active components that they contain. It exists also a certain number of databases focusing on NPs from plants used in traditional medicines on the African continent. Among those, the most famous and the most generalistic is AfroDB [110], although it is only accessible through the ZINC catalogues. The pan-African natural products library (p-ANAPL) also needs to be cited here, as it focuses on plants used in ATM and is available as the supplementary information if its publication [111]. Three datasets, AfroCancer [112], AfroMalariaDB [113] and Afrotryp [114], available as supplementary information of their respective publications link NPs from plants used in traditional medicines to their potential targets involved in the treatment of cancer, malaria and Trypanosoma. There are then country-specific and relatively small databases for NPs extracted from ATM plants, such as the Cameroon Medicinal Natural Products database (CamMedNP) [115], Central African Medicinal Plants database (ConMedNP) [116] and the Ethiopian Traditional Medicine Database (ETM-DB) [117].

##### Databases of drug-like natural compounds

Not linked, at least directly, to the traditional medicines, there is a lot of pharmacological research around the therapeutic properties of NPs, and these are compiled in the databases for drugs and drug candidates. In these databases, natural compounds are generally associated with a type of disease or molecular targets or receptors they interact with, and a rich description of their molecular and overall effects on the state of a patient or of a healthy person. The reference database in this category is DrugBank [118]. It latest version, which was greatly modified and curated compared to previous ones, contains over 10,000 drugs, among which 3732 are approved drugs and 200 approved drugs that have been produced by a living organism. In order to select only the latter, one needs to search for “nutraceuticals” in the search bar of the DrugBank website [119]. The previous version of Drugbank, 4.0 [120], contained over 8000 nutraceuticals, and they were added to COCONUT.

BindingBD [121] is an interesting database for pharmaceutical research as it contains measured binding affinities of proteins that are supposedly targets of drugs, with small drug-like molecules. Although it does contain NPs and their protein targets, they are not clearly distinguishable from synthetic drugs in this database.

The Novel Antibiotics Database [122], that is still surprisingly online, is not updated since 2003 and contains 5430 compounds of natural origin with an antibiotic activity that have been published in the Journal of Antibiotics between 1947 and 2003. However, no structure is available for download, only compound names, their activity and the organisms they were isolated from.

ChemIDplus [123] is a database part of the TOXicology DataNETwork and chemicals that have a relationship with diseases, environment, environmental health and poisoning. It contains rich metadata for each chemical, including its physicochemical properties but also its impact on health and environment. A simple search for “natural product” returns more than 9000 entries, it is however not possible to bulk download the results of the query.

The Herbal Ingredient Targets (HIT) [124] and the Herbal Ingredients in vivo Metabolism (HIM) [125] databases are two inter-connected collections of NPs from mainly (but not only) Chinese plants. Both are not accessible online anymore, but the structures of the NPs they contained are available on ZINC. They contained very extensive metadata on the molecular targets of the herbal active ingredients, their toxicity, a wide range of pharmacologically relevant molecular descriptors and their therapeutic effects. Unfortunately, this metadata is not available on ZINC and is probably lost.

There are several databases that focus on collecting information on NPs with anticancer properties and their mechanisms of action. The first one, NPCARE [126] contains over 6000 NPs from plants, marine organisms, fungi and bacteria with validated anticancer activities and contains extensive metadata. The website is available and seems updated but cannot be accessed sometimes, probably due to server failures on the maintenance side. The Indian Plant Anticancer Compounds Database (InPACdb) [127] is not available anymore but used to contain very broad information covering pharmaceutical and physicochemical properties of 144 NPs, cancer types and molecular targets. Fortunately, the data is still available on GitHub [128]. Another database, containing phytochemicals with anti-cancer properties is the Naturally Occurring Plant-based Anti-cancer Compound-Activity-Target (NPACT) database [129] is still maintained and accessible It contains 1574 manually curated entries with rich metadata on NPs and their therapeutical mechanisms on different types of cancer. The US National Cancer Institute also maintains and makes freely available a number of small (390 on average) natural compound datasets [130] that are selected as of interest in anticancer research and are currently undergoing tests in various research groups from the US NIH.

InflamNat [131] is a small (200 NPs) but well-curated dataset of NPs with anti-inflammatory activity. The dataset consists of NP structures, their type and origin and literature references, and is available as supplementary information for its publication.

BioPhytMol [132] is a manually curated database of natural compounds from plants that have an antibacterial effect. The database has over 2500 entries with very rich metadata, in particular regarding the plant species from which the compounds were extracted. The database is open and maintained but does not offer a bulk download option to be used to further analyses.

The last database in this section is the Open Source Malaria [133], which is a very nice project as it is a totally open-source collaborative project for anti-malarial drugs discovery that already encountered certain success [134]. Drug candidates tested in this project are often of natural origin, but as the focus of this database is to collect their effects, it is not always specified, so the content of OSM was not integrated into COCONUT.

##### Food

FooDB [8] is the reference database on chemical food constituents associated with extremely rich and diverse metadata. It is developed by the Wishart research group and supported by the Canadian Institutes of Health Research. In total it contains over 22,000 NPs and offers a convenient bulk download their structures.

BitterDB [6] collects bitter-tasting natural compounds associated with rich metadata on their receptors. However, it also contains synthetic molecules with a bitter taste, and in this database, it is difficult to separate them from the natural ones.

Phenol-Explorer [135] is a comprehensive database on polyphenol content in food. It currently contains over 800 phenol structures from over 400 foods. Data is derived from the scientific literature, and all data is associated with rich metadata and is available for download.

PhytoHub [136] is a database of dietary phytochemicals and the human and animal metabolites that derive from them. Over 1200 NPs from more than 350 foods are available in this resource, together with rich metadata and references to other chemical and spectral databases it, unfortunately, does not offer a bulk download for the moment.

The SuperSweet database [4] is a collection of various molecules, mainly from plant origin, but also synthetics that have a sweet taste. Their structures together with information on their number of calories, therapeutic uses and sweetness index are available. The database is still maintained but is not updated since 2011 and does not provide a bulk download of its content.

##### Toxins

A toxin is a substance that is toxic for one or more living organisms and that has a plant or animal origin. Despite this original definition, more and more resources on toxins also integrate molecules from non-organic origin massively present in the environment as they also have a harmful effect on the living organisms. For instance, Exposome-explorer [137] is a manually curated database of biomarkers of exposure to environmental and dietary factors, and it also contains these factors and their structures. A lot of the toxic environmental and dietary factors in it are from natural origin, but also, approximately half of the compounds in this database are not NPs, which is reasonable, as, for example, environmental pollution is anthropogenic. In the same way can be mentioned the T3DB [138], the toxin and toxin-target database, as it contains a number of toxins produced by the living organism but its focus is on synthetic toxins and how human metabolism reacts to them.

The biggest (over a 1000) database of animal toxins was the Animal Toxin Database (ATDB) [139], designed originally to collect toxin structures, origins and effects, but it is not available anymore at the URL provided in the publication. More specialized databases were also published, such as the International Venom and Toxin Database [140], the Snake Neurotoxin Database [141], the Mollusk Toxin Database [142] or the Scorpion Toxin Database [143]. Unfortunately, most of these databases were based on unformatted text and were lacking effective systems for data query, and none of them is not accessible anymore. It is also unknown if the data contained in these databases is lost or is still available in some generalistic resources.

The last in this section, the Toxic Plants—Phytotoxins Database (TPPT) [144], is accessible and is maintained and updated by the Agroscope in Switzerland. It contains over 1500 phytotoxins from Central Europe and offers high-quality metadata and a convenient bulk download.

##### Other

The two databases described next could not be fitted in any of the previous categories. The Carotenoids database [145] is a collection of NPs produced by a wide range of organisms and that share common substructures (polyene with possibly terminating rings) and properties as they are all yellow, orange or red pigments. Carotenoids produced by plants have particular importance for the nutritional value of the consumed food [146], but plants are not the only producers of this molecular type which is demonstrated in the Carotenoids database. This database is developed and maintained at the RIKEN institute. SuperScent [10] is a database of volatile compounds essential from an organic origin that can be scented by humans and animals. It contains over 2000 compounds with their structures and properties but does not offer any download and most of the compound pages are now working. This database is maintained at Charité Belin but is not updated since 2010.

#### Natural products by the geographic origin of producing organisms

There is a number of country-level efforts to catalogue the biodiversity of NPs in particular geographical zones, generally defined by country political borders. These databases are mainly plant-focused, but can also integrate NP produced by insects, by microorganisms and animal toxins. In this part, the databases are cited in the geographical order from West to East. The last part is describing collections of NPs from organisms in marine and ocean environments.

BIOFAQUIM [147] is a database published in 2019 and offers for full download over 400 unique NPs from plants, fungi and propolis from Mexican flora and fauna, the species from which the compounds were extracted and their geographical location. The Nuclei of Bioassays, Ecophysiology and Biosynthesis of Natural Products Database (NUBBEDB) [148] is the first NP library from Brazilian biodiversity. It currently contains over 2000 NPs, highly curated and good quality metadata and easy download of all or partial data. The UEFS dataset [149] is a collection of NPs isolated from Brazilian plants and maintained by the State University of Ferriera de Santana in Bahia, Brazil. The NPs in this collection have been published separately but there is no common publication nor public database for it, it is however accessible via ZINC.

Three databases contain NPs from the African flora and fauna. The Northern African Natural Products Database (NANPDB) [150] contains over 4500 NPs from plants, endophytes, fungi and bacteria. The database provides rich metadata, literature references, cross-references to major chemical databases and an easy bulk download. The South African natural compound database (SANCDB) [151] is very similar to NANPDB in its quality and contains over 600 NPs isolated from South African biodiversity. It is also possible to submit new molecules and to participate in the curation of the database. The Mitishamba database [152] contains 1100 NPs isolated from Kenyan plants. The database is still maintained but does not seem to be updated and it is possible to download data from it only by requesting an account.

ChemDB [3] and MAPS database [153] are two databases for natural compounds from Pakistani plants. Unfortunately, none of them is accessible anymore. VIETHERB [154] is a database published in 2018 with the aim of providing high-quality and literature-based data on herbs and active compounds from them. Despite the novelty of the database, it is not accessible anymore.

The oceans cover 71% of the surface of the Earth, therefore databases that collect NPs from marine organisms are expected to be broad, complex and cover a wide range of organisms. Unfortunately, the biggest repositories for marine NP structures are commercial (e.g. MarineLit [33] and DMNP [28] presented above). In the marine NP community, the major trend is to publish newly discovered molecules in specialised journals (such as the Journal of Natural Products [155] or Marine Drugs [156]) as images and rich textual description that are not, for now, easily machine-retrievable.

In the last 20 years, four databases containing structures of marine NPs and their metadata were published. Two of them are not accessible anymore: the Marine Compound Database (MCDB) [157] and the Marine Natural Product Database (MNPD) [158]. Both contained only a few hundreds of entries according to their respective publications but these were comprising rich metadata which is now lost. The Dragon Exploration System on Marine Sponge Compounds Interactions (DESMCI) [159] is still accessible but seems not to be maintained as the actual data, such as molecular structures and the corresponding metadata is not visible when one tries to access it. The Seaweed Metabolite Database (SWMD) [160] is the only one really maintained and it contains 1110 entries, with only 423 unique structures. Molecular structures in this database are annotated with the species of the algae that produce them, together with the geographical origin of the latter, biological activity of the compound and its physicochemical properties.

### Industrial catalogues

A lot of companies that are synthesizing and isolating chemical compounds offer a catalogue of their products, and in some cases, these catalogues also contain the structures and annotations. These catalogues are often cited in the scientific literature as sources of NP structures, therefore it was important to mention the most used catalogues in this review. Surprisingly, a non-negligible number of cited catalogues of NP structures are accessible only to clients, on-demand or to registered users. This is the case of the NP catalogues from Ambinter-Greenpharma natural compound library [161], ChemBridge diversity datasets [162] (their NP catalogue seems to be not available anymore), LOPAC1280 by Merk [163], Prestwick [164] and TargetMol [165]. Open NP catalogues are provided by the following: AnalytiCon Discovery [166], InterBioScreen [167], Indofine Chemical Company [168], Pi Chemicals Systems [169] and Specs [170]. The website of the latter is not offering the download of their NPs catalogue anymore, but a dataset is available on ZINC [171]. Note that only the most famous and cited in academic research are listed and more industrial catalogues for NPs exist.

### Problems

The biggest problem nowadays is that there are too many sources for NPs. A non-experienced researcher in NPs (and even a more experienced one) will just get lost in this variety and diversity of possible data sources. The next major problem is access to data and its maintenance. Indeed, a lot of publications point to a website that is not maintained anymore. This is the case of the majority of animal toxins databases, but also of a number of small regional or traditional medicine databases. In the list of NP sources presented in Table 1, over 20% are not maintained anymore or the access is intermittent. In some rare cases, the information on the NP structures is still recoverable via the ZINC database, but it is not the case of more modern databases and ZINC does not store any metadata from these collections, only the molecular structures encoded in SMILES. Also, the description and origins of the NPs (i.e. metadata), in addition to their structure are generally lacking, and it is especially the case in data aggregators that are nevertheless the most commonly used. This leads to cases where in silico screening reveals potentially interesting compounds but requires way more efforts and investigations to identify its origins and the way of obtaining it experimentally. Only 40% of NP databases offer an easy bulk download of molecular structures that they contain for further analyses with local tools. The quality of the molecular structures might also require additional attention and curation efforts. Indeed there are no standards for NP databases for a definition of stereochemistry, aromaticity or isotopes, which leads to a variety of possible versions of the same molecule.

This multiplicity of databases comes also from the publishing pressure on scientists, the infamous “publish or perish”. Nowadays, publishing a dataset or a database is a relatively easy publication and have the potential to generate a high number of citations. However, this trend generates a plethora of databases that are unmaintained beyond the publication time (like it is the case of VIETHERB [154] for example, published only 1 year prior to the writing of the present review and already not accessible anymore), despite the journals requirements to provide accessibility to the published datasets and databases for a number of years ahead.

## Comparison and analysis of the content of open NP databases

The 50 NP collections from which NP structures could be downloaded were analysed in order to evaluate their overlap in terms of molecular structures and coherence of their content. 19 physicochemical properties, such as molecular weight, NP-likeness [172, 173], logP, TPSA Efficiency, and Zagreb Index, were computed and their distributions are shown in an interactive graphic at https://npreview.naturalproducts.net. Due to the high number of databases to compare, a non-interactive would not be visible. Globally, the physicochemical properties of all datasets are comparable. The NP subset of Drugbank contains molecules that are less likely to be NPs, which can be explained by its high content in NP-derived drugs and the difficulty in dissociating the latter from synthetic ones. The average mass of all NPs in the assembled collection is of 454 Da, and the Spektraris and TCM@Taiwan databases contain the heaviest molecules: both contain molecules with an average of 612 Da. The logP is a lipophilicity measure commonly used in analytical chemistry; the more it is positive, the more lipophilic is the compound and the more negative, the more hydrophilic. Here, the logP was computed with two algorithms, AlogP and XlogP available in the CDK [174]. In general, NPs tend to be lipophilic, which allows them to have higher membrane penetration, but all datasets also contain in lesser amounts, hydrophilic molecules. CarotenoidsDB and the SeaWeed Metabolites Database outstand from others with their very lipophilic content. On the other side, ReSpect contains more hydrophilic molecules than other datasets.

**Fig. 1:**
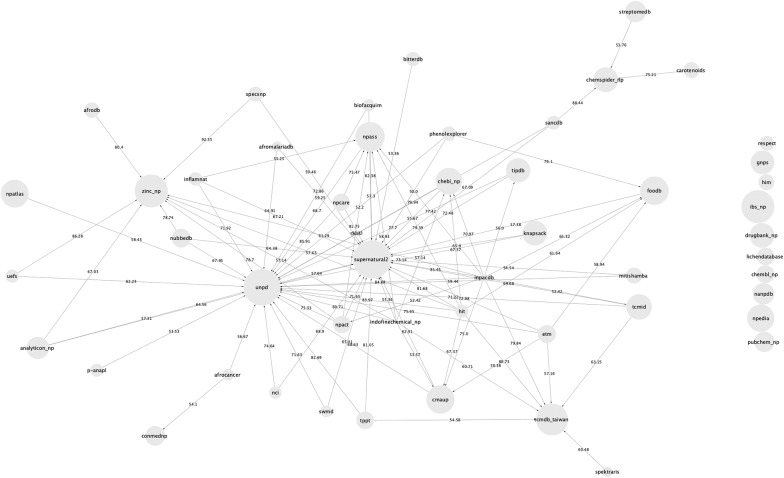
Network of content similarity between the 50 open natural products databases. The network is directed, and there is an arrow from database A to database B if more than 50% of molecules in database A are also present in database B. The interactive version of this network is available at https://npreview.naturalproducts.net

The overlap in terms of molecular structures between the databases was also calculated and is presented in Fig. 1 and in Additional file 1: Table S1. In Fig. 1, which represents a network of overlap between databases, there is a directed edge between database A and database B if more than 50% of the unique molecules from database A are present in database B. An interactive version of this network, where the user can change the percentage of similarity between databases to display is available at https://npreview.naturalproducts.net. It should be noted that 40 of the 50 open NP databases have an overlap of at least 50% with at least one other open database. Except for the Lichen Database, all datasets share at least 10% of their compounds with at least one other open dataset.

In the majority of the databases, stereochemistry is defined for at least some of their content. Only three databases, TCMid, ReSpect, and NPCARE don’t have any stereochemistry defined for any of the molecules in them. The fraction of NPs with stereochemistry in each database is accessible in Table 1. On average in the open NP databases, more than 50% of the molecules have a defined stereochemistry. When a 2D molecular structure is present in two databases and stereo information was elucidated, in general, open databases tend to agree on the latter. Doing a pairwise comparison between databases on their overlapping content, pairs of databases tend to agree on the stereochemistry, in on average 70% of NP than they share. The whole list of pairwise agreement between databases on the stereochemistry of their overlapping molecules can be found on FigShare (10.6084/m9.figshare.11926047.v2).

**Fig. 2:**
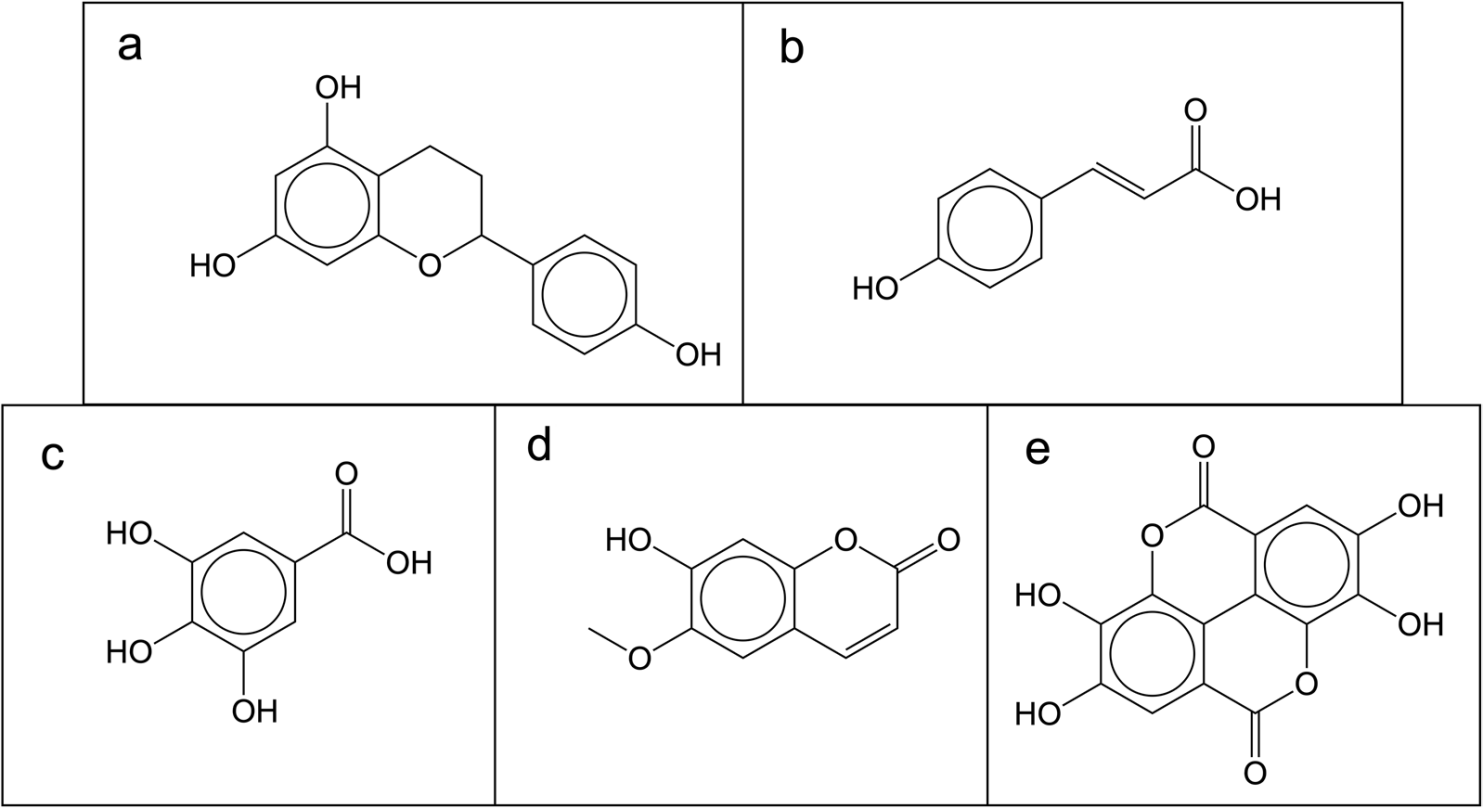
Most frequent molecules in open databases. **a** Common biggest substructure in the top 5 most frequent molecules, found in 34 out of 50 open databases. **b** Coumaric acid; **c** gallic acid; **d** scopoletin; **e** ellagic acid

Five NPs are found in 34 of these 50 databases: apigenin, quercetin, kaemferol, catechin and naringenin. Interestingly, belong all to the flavanol group, part of the flavonoids family and share a common skeleton (Fig. 2a) with only differences in hydroxy groups. In the top ten most frequent molecules in open databases, in addition to more flavonoids, there is also coumaric acid (Fig. 2b), gallic acid (Fig. 2c), scopoletin (Fig. 2d) and ellagic acid (Fig. 2e). According to the literature, all these compounds are well-known plant products, however, most of the flavanols, coumaric acid and scopoletin are also present in the bacterial NP database, StreptomeDB.

## COlleCtion of Open NatUral producTs (COCONUT)

In its current version, COCONUT contains 411,621 unique molecules, unified on the stereochemistry-free InChi keys, that were collected from 50 open and accessible NP databases, listed in Table 1. This number is big, as this dataset still needs to undergo a curation process, as, despite their claims, some of the NP collections do not contain only natural compounds. 27.9% of molecules in COCONUT do not have stereo centres defined in any of the databases where they have been collected from. Among the latter, 57.7% (66,374 unique molecules) have truly no stereocenters, and the remaining 48,611 NPs have at least one stereocenter, but this information is not provided.

50% of the unique molecules have only one stereochemical version of their 3D structure, and 22.1% have more than one. The latter could be different valid stereoisomers of the same base constitution or errors in the databases. Addressing those errors will be subject of future curation of COCONUT. When a 2D molecule has several possible 3D structures, these can originate from the same public database, where stereochemistry is precisely defined, but also from different databases. Note that unknown NP structures or mixtures are not included in COCONUT. The collection is available as a MongoDB dump and a CSV file on Zenodo (10.5281/zenodo.3547718) and a user-friendly web interface to browse it is under development. The aim of COCONUT is to make the NP-related data as FAIR as possible.

## Discussion

There are currently 123 data collections of natural products (NPs) that have been published and cited in the scientific literature between 2000 and 2019. Only 50 of them are open access or have their content accessible (in ZINC for example) and among them, the overlap of their content is significant, as 40 of these datasets share at least 50% of the compounds they contain with at least one other dataset.

There are several aggregators, such as the ZINC catalogue for NPs, SuperNatural II and UNPD (not maintained anymore), but they do not cover the entire space of known NPs and do not allow submissions of newly discovered compounds.

There is a need for an aggregator database for NPs, that will be commonly recognized, well organized and allowing an easy submission of newly found molecules, like it is the case for UniProt for proteins.

## Conclusions

Natural products are important molecules for medical, chemical and social research. There is no, for now, any universal, community-accepted database for NP discovery, screening and dereplication. Instead, there is an extremely high number of very diverse databases and datasets, not all maintained or open access in 2020, which represents a serious loss of knowledge. There is a need for a unified universal repository for NPs, to avoid the unnecessary duplication of online resources and facilitate NP research. For the purpose of this review, a COlleCtion of Open Natural prodUcTs (COCONUT) has been assembled, analyzed and made available in Zenodo (10.5281/zenodo.3547718). A web interface is currently under development for user-friendly querying, exploration and download of the known open NP space. In the future, the annotations of the molecules contained in COCONUT will be improved, in particular, systematically linking the compound to the first publication where it was described and to the organisms that synthesize it.

## Materials and methods

All databases in Table 1 were downloaded in July and September 2019. Molecular structures were processed with CDK 2.3 and, when available, annotations were parsed with Java (code available on GitHub https://github.com/mSorok/COCONUT). Resulting original and non-redundant collections of NPs are stored in a MongoDB database, available as a dump on Zenodo (10.5281/zenodo.3547718). Redundancy was eliminated based on InChi Keys, computed without stereochemistry (JNI-inchi option of the InChi generator set to “Snon”, “ChiralFlagOff” and “AuxNone”). Stereochemistry was not taken into account during this unification step as it is encoded differently between some databases and there are databases where it is not encoded at all. The overlap between databases in terms of similar stereochemistry was also performed with CDK 2.3. All network representations of overlaps between databases are made with Cytoscape [175]. Plots and comparative analyses made with Python and the Plotly and Dash libraries. The code for the interactive plots is available on GitHub at https://github.com/mSorok/NPDBReviewDash.

## Supplementary information

**Additional file 1.** Overlap (in percent) of compound content between open natural products databases.

## Data Availability

Data and software are freely available under the MIT license. The source code for data processing can be freely obtained from GitHub (github.com/mSorok/COCONUT), the COCONUT data is available on Zenodo (10.5281/zenodo.3547718). The interactive application for natural products exploration is available at https://npreview.naturalproducts.net/ and the code is available on GitHub (https://github.com/mSorok/NPDBReviewDash). The table compiling all assembled natural products resources is available on FigShare (10.6084/m9.figshare.11926047.v2).
